# A Review on Pulsed Laser-Based Synthesis of Carbon and Graphene Quantum Dots in Liquids: From Fundamentals, Chemistry to Bio Applications and Beyond

**DOI:** 10.1021/acs.jpcc.5c01343

**Published:** 2025-06-02

**Authors:** Francis Rey U. Cortes, Eva Falomir, Carlos Doñate-Buendía, Gladys Mínguez-Vega

**Affiliations:** † Group of Optics-UJI (GROC-UJI), Institute of New Imaging Technologies (INIT), 16748 Universitat Jaume I (UJI), 12071, Castellón, Spain; ‡ Department of Inorganic and Organic Chemistry, 16748 Universitat Jaume I (UJI), 12071, Castellón, Spain

## Abstract

The constantly growing interest in zero-dimensional carbon-based nanomaterials such as carbon quantum dots (CQDs) and graphene quantum dots (GQDs) as the vital components in advancing various bio-related, catalysis, and energy-relevant applications has inspired nanotechnology research centered mainly on their synthesis and modifications for the betterment of their exceptional features including their strong and tunable fluorescence. Among the multitude of synthesis approaches in fabricating CQDs and GQDs, laser-based synthesis in liquids, such as pulsed laser ablation in liquids (PLAL) and pulsed laser fragmentation in liquids (PLFL), has emerged as a more beneficial technique owing to its versatility, flexibility, green synthesis process, and ease of scalability. With the modern trend of employing this method for CQDs and GQDs synthesis, this review article will revisit the foundation of laser synthesis in liquids, starting from its fundamental mechanism of nanoparticle formation to the effect of different variables such as laser parameters (e.g., laser energy, laser wavelength, frequency), chosen liquids, and the starting carbon material to the final attributes (morphological, optical, and surface) of CQDs and GQDs. In this paper, we will also address the different post-laser treatments, such as modifications and conjugation, and how they affect the properties of CQDs and GQDs. We will also emphasize the diverse applications of laser-synthesized CQDs and GQDs, ranging from bioapplications to beyond bio-related applications. This article hoped to provide practical insights for researchers to develop further laser synthesis in liquids to produce carbon-based nanomaterials such as CQDs and GQDs and their applicability to other applications.

## Graphical abstract

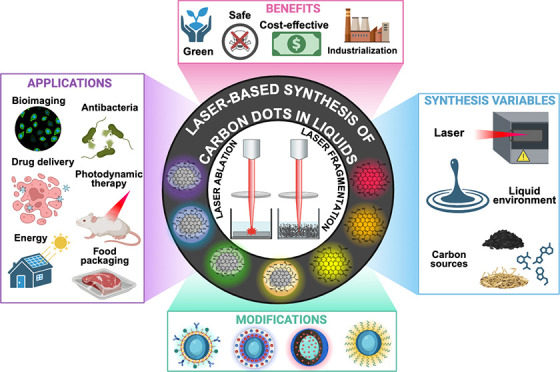

## 1 Introduction

As one of the newly discovered foundations of carbon-based nanomaterials, carbon quantum dots (CQDs) and graphene quantum dots (GQDs) are emerging classes of zero-dimensional (0D) materials that have gained tremendous interest owing to their vast array of practical applications in biomedicine, catalysis, and energy-related applications. The capability of these materials to revolutionize these applications stems from their exceptional properties, including excellent fluorescence and conductivity, chemical inertness, low or no cytotoxicity, high biocompatibility, and outstanding photostability.^1–6^ These distinct properties proved their advantage in various fields, from *in vitro* and *in vivo* bioimaging,^7–10^ bio- and chemical sensing,^9,11,12^ photothermal and photodynamic therapy (PTT/PDT)^13–15^ to chemotherapeutic drug delivery system^16–18^ and even photocatalysis^19,20^ and light-emitting diodes (LED).^21^ Among the mentioned characteristics of CQDs and GQDs, their strong and tunable photoluminescence (PL) emission is one of the striking properties that has encouraged further investigation among the scientific community in recent years. In particular, this intrinsic fluorescence attribute of CQDs and GQDs may offer a potential alternative to conventional semiconductor quantum dots (SQDs) and organic fluorescent dyes (OFDs). Although the SQDs undeniably possessed exceptional PL, as proven by being the awardee of the Nobel Prize in Chemistry last 2023,^22^ their high toxicity, due to heavy metal composition, substantially interfered with their full capability to use in biomedical applications.^23,24^ On top of that, the fluorescence of SQDs is readily prone to photobleaching and PL blinking, which can influence their performance, especially in bioimaging and fluorescence sensing.^25,26^ Photobleaching has also become a significant problem faced by OFDs, which can significantly affect their vital function in bioassays, bioimaging, and diagnostic purposes.^27^ Such indisputable limitations imposed by SQDs and OFDs are uncommon characteristics of CQDs and GQDs. Thus, the possibility of employing these carbon-based nanoparticles (NPs) as substitutes for them is no longer beyond reach.

To fully outperform their semiconductor QDs and organic fluorophore counterparts, the current challenges of CQDs and GQDs, such as their generally low fluorescence yields, especially in PL emission in deep red/near-infrared regions, must be carefully addressed first. Due to the near-infrared window in biological tissue, an enhanced fluorescence yield in those regions is necessary for bio applications. In line with this, several synthesis methods for preparing CQDs and GQDs were explored with numerous modifications and techniques to solve these issues, aiming for simplicity, cost-effectiveness with significant production rates, and green synthesis.^28–30^ Similar to other nanomaterials, the synthesis routes of CQDs and GQDs can be broadly divided into ***top-down*** and ***bottom-up*** approaches.^4,6^ The common bottom-up approaches for CQDs and GQDs synthesis include electrochemical carbonization,^9^ microwave irradiation,^31^ thermal decomposition,^32,33^ and hydrothermal/solvothermal treatment.^34,35^ On the other hand, top-down approaches commonly involve electrochemical oxidation,^36,37^ laser-based treatment,^38,39^ chemical oxidation,^40,41^ arc-discharge treatment,^42,43^ and ultrasonication.^44,45^ Amidst the plethora of synthesis routes to produce CQDs and GQDs, the laser-based synthesis method shows great potential to open a new avenue of non-conventional techniques^46^ in tailoring different kinds of CQDs and GQDs of varying properties depending on their application.

Various laser-based techniques are being employed in the fabrication of not only CQDs and GQDs but nanoscale materials in general as well, namely laser vaporization,^47^ laser deposition,^48^ laser-chemical vapor deposition,^49^ laser pyrolysis,^50,51^ and the like.^52^ Among them, “***laser synthesis and processing of colloids***” (LSPC), alternately called laser synthesis in liquids^53,54^ created a notable impact in nanotechnology due to its versatility and ease of scalability to synthesize high-purity nanomaterials in a controlled liquid environment. Figure 1 shows the four methodologies within LSPC, namely, (1) *laser ablation in liquid* (bulk target as a target material),^55–57^ (2) *laser fragmentation in liquid* (micro powder or colloidal suspension as a target material),^58–60^ (3) *laser melting in liquid* (micro powder or colloidal suspension as a target material),^61–63^ and (4) *laser reduction in liquid* (molecular precursors in solution).^64,65^ LSPC primarily exploits a high-energy pulsed laser beam to produce, extract, and modify NPs from a target material. The liquid effectively collects the produced nanomaterials and, simultaneously, is the central basis of *in situ* chemical reactions, which could lead to doping,^66–68^ surface functionalization,^69,70^ oxidation, and even defect formation. Additionally, the liquid guarantees a safe space for synthesizing NPs without posing any threat to the health of an experimenter, such as inhalation, compared to laser synthesis in air.^54^

**Figure 1:**
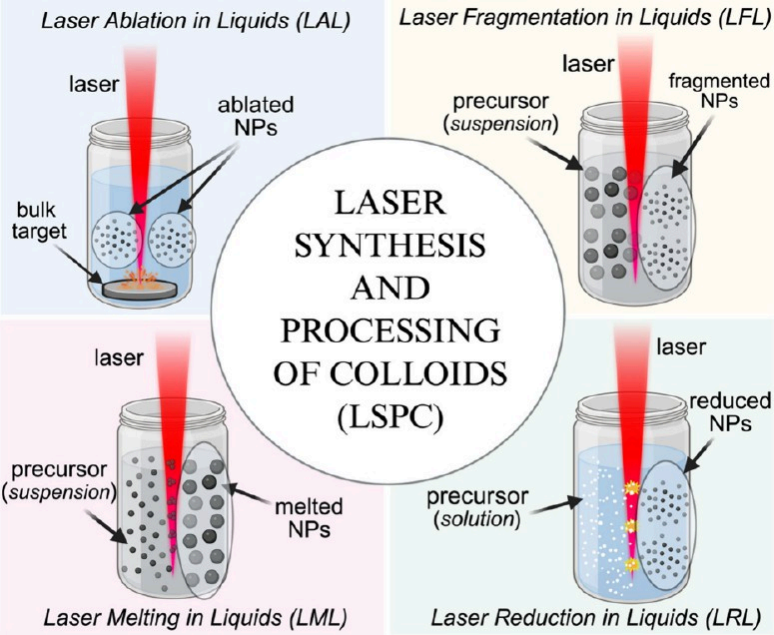
General techniques of the LSPC-based synthesis of nanomaterials.

Historically, the integration of laser to produce nanomaterials dates back to the groundbreaking works of Patil et al. in 1987 ^71^ on the successful synthesis of iron oxide in water using a ruby laser and of Fojtik and Henglein in 1993 ^72^ on the laser synthesis of gold, nickel, and carbon colloidal solutions at different solvents (water, 2-propanol, and cyclohexane). Since then, LSPC has drawn a considerable amount of attention as an efficient method to fabricate a vast collection of high-purity nanomaterials ranging from carbon-based materials,^10,73^ metal NPs,^74,75^ semiconductor QDs,^76^ oxides,^77^ non-oxides,^53^ chalcogenides,^78^ and composites nanostructures^79^ with diverse and improved chemical, optical, electronic, electrical, magnetic and morphological properties. Moreover, LSPC was the method employed by Sun et. al.,^80^ who formally coined the term “*carbon dots*” in 2006 following their accidental discovery in 2004 during the purification of carbon nanotubes by Xu et. al. From then on, research on LSPC-based CQDs and GQDs has significantly expanded, leading to one-step surface modification, tuning of the properties through laser parameter adjustment, and, most importantly, the CQDs and GQDs promising potential in biomedical applications. Figure 2a shows the historical timeline and development of LSPC-based synthesis of CQDs and GQDs.^10,71,72,80–96^

**Figure 2:**
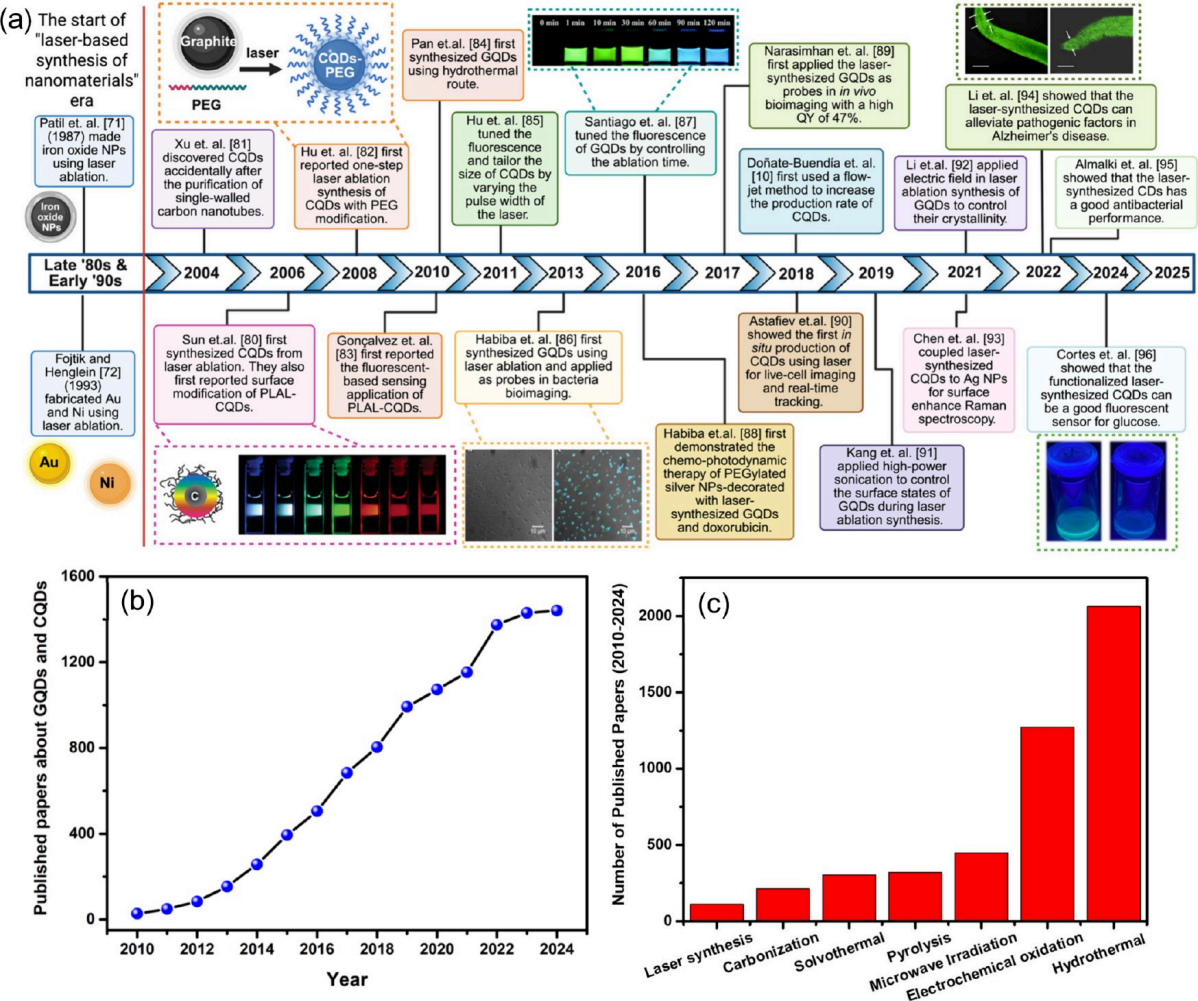
(a) Historical timeline and development of LSPC-based synthesis of CQDs and GQDs starting from the pioneering works of Patil et al. and Fotjik and Henglein. Figures from Sun et. al. were reprinted with permission from ref 80. Copyright 2006 American Chemical Society. Figure from Santiago et. al. was reprinted with permission from ref 87. Copyright 2016 Royal Society of Chemistry. Figure from Habiba et. al. was reprinted with permission from ref 86. Copyright 2013 Elsevier Ltd. Figure from Li et. al. was reprinted with permission from ref 94. Copyright 2025 Elsevier B.V. The whole timeline and schematic figure from Hu et. al. were created in BioRender.com. (b) Number of published papers on CQDs and GQDs and (c) Comparison of published papers on laser synthesis of CQDs and GQDs to other conventional approaches based on the *Scopus database* between 2010 and 2024.

Despite their advantages in producing CQDs and GQDs, LSPC has been outnumbered by other established synthesis routes. Although the number of published papers regarding CQDs and GQDs is increasing year by year (Figure 2b), no more than 2% of the total published papers (2010-2024) is based on the laser-synthesized CQDs and GQDs according to the search filter keywords (from the Scopus database): “*carbon quantum dots*”, “*graphene quantum dots*”, “*laser ablation*”, and “*laser synthesis*”. Figure 2c shows the comparison of published papers between the laser-based technique and other conventional methods for the production of CQDs and GQDs from the Scopus database within the time frame between 2010 and 2024. Regardless of this, there are some excellent reviews about LSPC-based synthesis of CQDs and GQDs, such as the review from Yogesh et al. on laser synthesis of all derivatives of carbon nanomaterials (carbon nanotubes, GQDs, CQDs, polyynes, and nanodiamonds)^73^ and Amans et al. on the laser synthesis of nano-carbon allotropes materials such as nanodiamonds and related materials.^97^ This review aims to provide the status and progress of laser-based synthesis research on CQDs and GQDs, emphasizing their modifications, properties, and applications. There are four sections in this review: the first section introduces the structural and photoluminescence properties of CQDs and GQDs and the advantages and drawbacks of the laser-based synthesis technique; the second section explains the different PLAL and PLFL experimental configurations, the fundamental mechanism of NPs formation using PLAL and PLFL, and the effects of various variables (laser parameters, solvent, carbon precursors) to the final properties of CQDs and GQDs; the third section focuses on the different applications of laser-synthesized CQDs and GQDs from bio applications to industry. Lastly, we addressed and presented the challenges, outlook, and future not only of this technique but also of CQDs and GQDs themselves.

### 1.1 Structural and PL Properties of CQDs and GQDs

#### 1.1.1 Structural Differences between CQDs and GQDs

Carbon dots (CDs) are a collective term referring to a variety of fluorescent carbon nanomaterials, which include GQDs, CQDs, and carbonized polymer dots (CPDs) according to their structure and properties (Figure 3a). CQDs and GQDs belong to the general category of CDs where their structural and chemical properties differ and usually mislead. The slight differences in structure and morphology create a boundary between them, which leads to their uniqueness and distinction. CQDs are quasi-spherical NPs, typically with a diameter of less than 10 nm. They are comprised mainly of a sp^2^-hybridized spherical carbon core arranged in the same way as multi-layer graphene structures and a disordered sp^3^-hybridized carbon matrix as the shell covering the carbon core composed of various chemical moieties on their surfaces such as carboxyl (−COOH), carbonyls (−C=O), hydroxyls (−OH), nitro (−NO_2_), and amino groups (−NH_2_) as shown in Figure 3b.^6,29,98,99^ In addition, the carbon core of CQDs can be a mixture of sp^2^/sp^3^-hybridized carbon originating from the structural defects within the core.^100,101^ On the contrary, GQDs are stacked nanosized graphene fragments with no more than five graphene layers and have a lateral size usually less than 100 nm.^99,102–104^ GQDs have a strong sp^2^-hybridization character and possess a disk-shaped morphology with various functional groups at the edges or within the interlayer spacing.^102,105–107^ In contrast with the spherical-like morphology of CQDs, GQDs are also in quadrate, hexagonal, or even triangular forms.^108,109^ Other than that, GQDs have a high degree of crystallinity compared to CQDs, which can be a crystalline and amorphous mixture.^7,106^ The starting materials, synthesis conditions (e.g., temperature and reaction time), and the synthesis methods can affect the composition of the CQD’s surface, the interlayer defects, and the GQDs edges.^99,110–112^ Moreover, the synthesis conditions can also dictate the formation of either CQDs or GQDs from the same precursor. For instance, Bokare et. al.^113^ demonstrated that GQDs are likely formed at a higher temperature during the chemical oxidation process using charcoal powder as the precursor. Furthermore, Kang et. al.^114^ also revealed that at a specific laser threshold energy during laser fragmentation synthesis of carbon nanotubes in ethanol, highly crystalline GQDs are formed. However, below this threshold, the synthesis leads to the formation of amorphous CQDs. To visualize the differences between CQDs and GQDs, Figures 3b and 3c show their typical composition and structure from top and side views, respectively. The differences might seem negligible since both have a structured layer of graphene in their cores and functional groups attached to them.^115,116^

**Figure 3:**
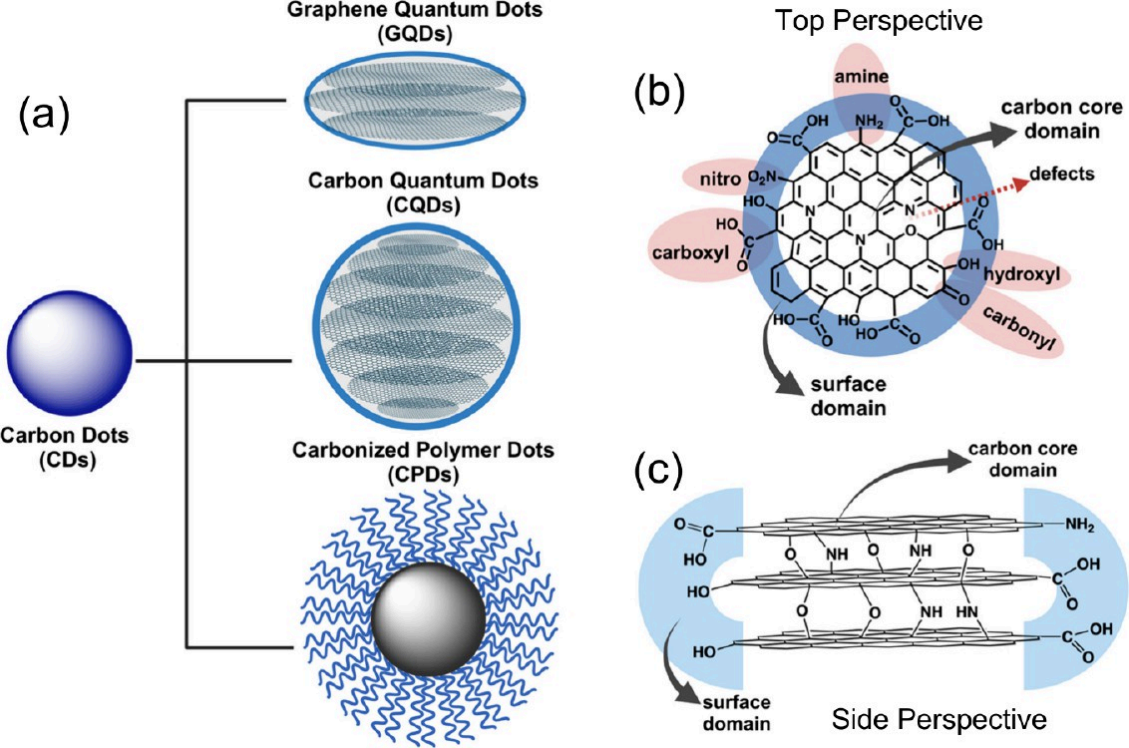
(a) CDs can be classified as GQDs, CQDs, or CPDs and structural representation of CQDs or GQDs when viewed at the (b) top and (c) side. Created in BioRender.com.

In addition, CPDs are another class of carbon dots that are polymer and carbon hybrid structures. Their core can be composed of highly crosslinked polymer or tiny carbon clusters surrounded by polymer, and their surface comprises abundant polymer chains or functional groups.^117,118^ This work does not provide an in-depth review of the CPDs. However, one can find noteworthy reviews centered on CPDs, such as the reviews from Xia et al.^117^ and Tao et al.^118^ With the difference between CQDs and GQDs defined, the general term CDs will be employed all over this review, unless otherwise specified.

#### 1.1.2 PL Properties of CDs

PL is a phenomenon in which a molecule absorbs light, promoting an electron to a higher excited state and then emitting a photon (fluorescence) as the electron returns to the lower energy state. PL is one of the most appealing properties of CDs because of its dependency on the excitation wavelength; that is, the emission peaks vary based on the excitation wavelength. Typically, the PL emission wavelength of CDs is longer than the excitation source (down-conversion fluorescence).^28,30,116,119^ The mechanism of this unusual behavior is yet to be uncovered and has primarily been a topic of debate up to the present time. Although there have been various theories regarding the PL of CDs, unfortunately, to this day, there is no consensus on which mechanism will unify and fully decipher the origin of their PL without any ambiguity. Nonetheless, there are three generally accepted mechanisms to elucidate the PL of the CDs such as (1) ***quantum confinement***, (2) ***surface states***, and (3) ***synergistic effect.*** ^116,120–124^

The quantum confinement effect (QCE), commonly known as the “*size effect*”, is one of the established PL mechanisms that also governs the fluorescence mechanism of conventional SQDs. As the size of the CDs decreases, the PL emission shifts to a shorter wavelength. This phenomenon correlates with the band gap between HOMO (highest occupied molecular orbital) and LUMO (lowest unoccupied molecular orbital), in which the larger size has a smaller energy gap corresponding to the longer emission wavelength^116,121,122^ (Figure 4a). In this case, the size refers to the sp^2^ graphene domain in the carbon core as the acting emission center.^116,125,126^ Yuan et al.^127^ prepared five CQDs synthesized by solvothermal treatment of citric acid and diaminonaphthalene. They found that the emission wavelength of CQDs strongly correlates with their size. Thus, they are also referred to them as band gap fluorescent CQDs (BF-CQDs). Figure 4b shows that the blue-, green-, yellow-, orange-, and red-BF-CQDs have average sizes of 1.95 2.41, 3.78, 4.90, and 6.68 nm, respectively. In addition, their fluorescence spectra also exhibited a red shift of emission peaks as the CQDs became larger. Moreover, Li et al.^128^ also obtained CQDs of different particle sizes and observed the PL emission dependence on the size of the CQDs: 1.2 nm CQDs emitted at UV region (350 nm), 1.5-3 nm CQDs emitted at the visible region (400–700 nm) and relatively large CQDs with a size of 3.8 nm emits at the near-infrared region. Figure 4c shows the fluorescence of the prepared CQDs under a UV lamp with their corresponding red-shifted fluorescence spectra, as shown in Figure 4d. To prove the size-dependent fluorescence, they measured the band gap (eV) of each CQD and found that the band gap decreases as the size increases (Figure 4e), which perfectly coincides with the size-dependent fluorescence schematics, as previously shown in Figure 4a. According to this mechanism, the excitation-dependent fluorescence of CDs originates from their size heterogeneity. CDs of varying sizes possess different band gaps, which lead to emission at distinct wavelengths depending on the excitation source. This broad size distribution is often attributed to poorly controlled synthesis conditions and the lack of an effective separation process. In the absence of any size separation process, Bhattacharya et al.^129^ confirmed that the excitation-dependency of CD fluorescence arises primarily from the inhomogeneous size distribution. While the QCE model has been widely adopted to explain this behavior, it is limited since some CDs display size-independent PL. This suggests that there must be another contributing factor to explain the excitation-dependent PL of these CDs other than their size, such as their surface composition (discussed below).

**Figure 4:**
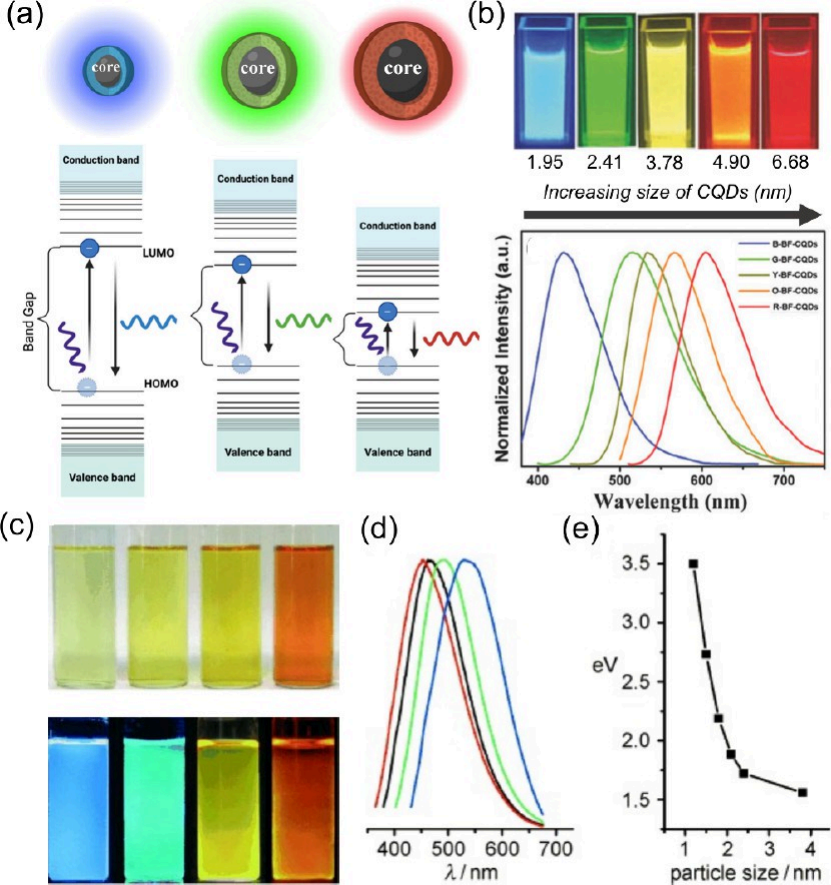
(a) Illustration of CDs fluorescence originating from the quantum confinement effect (Created in BioRender.com), (b) Fluorescence of prepared CQDs with increasing size and their corresponding fluorescence spectra (Reprinted with permission from ref 127. Copyright 2016 John Wiley and Sons, Inc.), (c) CQDs under ambient light and UV lamp with their (d) corresponding fluorescence spectra, and (e) band gap relation to their size (Reprinted with permission from ref 128. Copyright 2010 John Wiley and Sons, Inc.).

Another proposed mechanism that deviates from the size effect is the dependence of PL on the surface state of the CDs. Other than the carbogenic core as the emitting center, as stated by the QCE effect, surface states or surface traps also play an essential role in the PL of CDs. The surface groups provide an energy state that captures electrons during their relaxation. Increasing the surface groups and varying their composition produced multiple energy states, leading to a red-shift emission (Figure 5a). Herein, surface traps are a generalized term to include surface defects, functional groups (oxygen- and nitrogen-related), polymer passivation, and edge states.^116,121,125,130–132^ Yang et al.^133^ obtained full-color emission CQDs by controlling the surface state composition using different solvents, namely, formamide (FOR), ethyl acetate (EA), ethanol (EtOH), acetone (Ac), and water, using m-phenylenediamine and phosphorous acid as the starting precursors. They found that the prepared CQDs had approximately the same average size. Still, their corresponding fluorescence (under UV lamp) and fluorescence spectra showed a red-shift emission (Figure 5b). The synthesized CQDs in FOR, EA, EtOH, Ac, and water exhibited blue (B-CQDs), green (G-CQDs), yellow-green (YG-CQDs), yellow (Y-CQDs), and red (R-CQDs) color emission. In addition, they found that the amount of C=O content in the B-CQDs is significantly higher than that in R-CQDs. Thus, they concluded that surface states dominate their fluorescence rather than the size effect. They attributed the red-shift emission to the increase of the C=O content on the surface of CQDs, while the blue-shift emission is from the presence of pyridinic nitrogen. Ding et al.^134^ synthesized CQDs from urea and p-phenylenediamine via the hydrothermal method. By increasing the degree of surface oxidation, the fluorescence of CQDs significantly changes (Figure 5c). The increase of oxygen-containing functional groups (−COOH) can be the underlying reason for the red-shifted emission since all of the prepared CQDs have an average size of approximately 2.6 nm. Figure 5c also shows that increasing their surface oxidation leads to an increase in surface energy states, thereby reducing the band gap between LUMO and HOMO. Following this mechanism, the excitation-dependent PL characteristic of CDs primarily originates from the various surface traps that act as emissive traps. Owing to the heterogeneity of their surface composition, each surface state exhibits distinct energy levels with fluorescent emission depending on the excitation wavelength. For instance, Li et. al.^135^ measured the energy band gaps of the different functional groups on CQDs prepared from citric acid and urea. They found that CQDs with −COOH exhibited a band gap of 0.94 eV, while CQDs with −OH showed a band gap of 1.12 eV. These variations in band gap energies contributed to their excitation-dependent fluorescence behavior.

**Figure 5:**
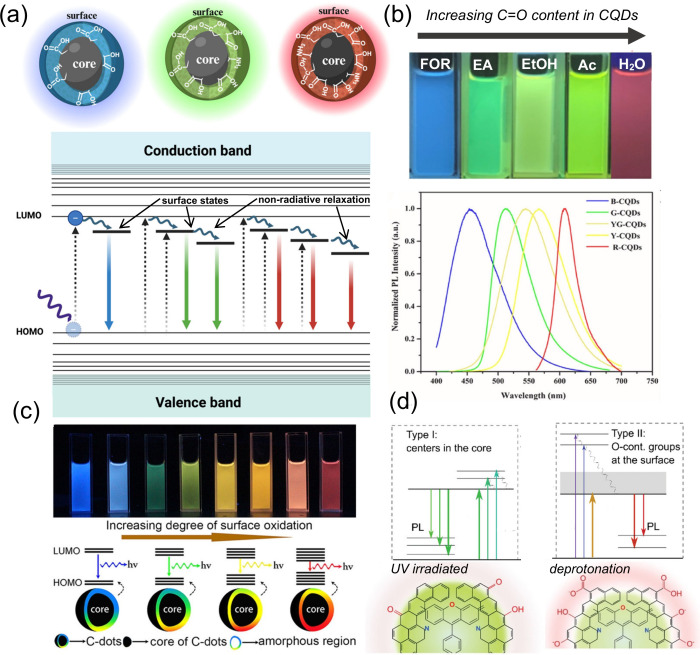
(a) Illustration of CDs fluorescence originating from the surface state effect (Created in BioRender.com), (b) Fluorescence of prepared CQDs at different solvent and their corresponding fluorescence spectra (Reprinted with permission from ref 133. Copyright 2022 Elsevier B.V.), (c) CQDs under UV lamp and the band gap illustration from the degree of surface oxidation (Reprinted with permission from ref 134. Copyright 2016 American Chemical Society), and (d) synergistic effect of carbon core and surface state emission (Reprinted with permission from ref 136. Copyright 2023 John Wiley & Sons, Inc.).

When the two PL mechanisms (QCE and surface state effect) failed to fully explain the PL observations individually, the synergistic effect is a model in which the QCE and surface state effect combined to elucidate the PL of CDs.^116,120^ Zhang et al.^136^ revealed that there were two emission centers of multi-color emissive CQDs synthesized from the citric acid in formamide. Type I emission center is from the inner sp^2^-domains (carbon core), while the Type II emission center is coming from the oxygen-containing surface groups attached to the surface (Figure[Fig fig5]d). They found that after constant UV-irradiation of CQDs, green/yellow emission is photostable and more pronounced. In contrast, the red emission decreased in intensity. After the solvent environment of the CQDs is changed, the red emission increases due to the deprotonation of the oxygen-rich surface while degrading the green/yellow emission. They conclude from these findings that the red emission originated from the Type II emission center, while the green/yellow emission is from the Type I emission center. Furthermore, Yu et. al.^137^ demonstrated that the multi-color emission bands of CDs synthesized via one-step microwave method using amino acids are related to different mechanisms within the CDs. They observed that the two emission peaks centered at 305 and 355 nm originated from the electron transitions within the carbon core, specifically from intrinsic C (C=C/C–C) and graphitic N-related impurity, respectively. Meanwhile, the peaks at 410, 445, and 500 nm are the result of surface-related electronic transitions involving pyridinic N, amino N, and C=O, respectively. Additionally, Dhenadhalayan et. al.^138^ showed that the multi-emissive sites of citric acid-derived CDs are the result of the coordination between the carbon core and surface state. Specifically, the fluorescence below 400 nm is caused by the carbon core, while the emissions above 400 nm arise from the surface functional groups conjugated on the carbon core. Thus, understanding the synergy between the carbon core and surface states is one of the key factors underlying the excitation-dependent behavior of the CDs.

Apart from their conventional fluorescence (down-conversion fluorescence), another characteristic of CDs is their ability to display up-conversion fluorescence. In contrast with down-conversion fluorescence, this kind of fluorescence emits wavelengths that are shorter than the excitation source. Cui et. al.^139^ showed that the up-conversion fluorescence of CDs is attributable to a nonlinear process known as multiphoton absorption where the CDs absorb two or more photons. This results in the promotion of the electron to a higher excited state, which then relaxes by emitting a high-energy photon. Another mechanism of CDs up-conversion fluorescence is based on the anti-Stokes transition model, where a low energy photon excites a *π*-electron at the intermediate energy level to LUMO then relaxes into *σ* orbital (HOMO) by emitting high-energy photon.^29,116,124^ However, the existence of up-conversion fluorescence in CDs remains a subject of debate, whether this phenomenon is truly an inherent property or merely artificial. Wen et. al.^140^ recently showed that the up-conversion of CDs could originate from the monochromator of the spectrophotometer itself. Thus, proper filters or thorough tests must be carefully performed to confirm CD up-conversion fluorescence.

In summary, the fluorescence mechanisms such as size effect, surface traps, and synergistic effect (carbon core and surface state) are the basic mechanisms that unravel the CD’s fluorescence and its dependency on the excitation wavelength (excitation-wavelength PL). The three mentioned PL mechanisms are based solely on integrating PL effects from carbon core and surface effects, not including doping effects, solvation effects, and external factors such as temperature, pressure, and pH.^116,141–144^ Although they are not flawless, they can explain the fundamentals of the PL of CDs. This review does not cover several theories, but readers can find them in other PL mechanism-focused literature (e.g., more insights on up-conversion fluorescence, the existence of excitation-independent PL, and phosphorescence of CDs) such as from Ai et al.^116^ and Gan et al.^120^

### 1.2 Advantages and Disadvantages of Laser-Based Synthesis of CDs over Alternative Methods

There are two main synthesis approaches for CDs: *bottom-up* and *top-down* approaches (Figure 6). The former involves the carbonization, assembly, or dehydration of small carbon-rich molecules and polymers through electrochemical carbonization, microwave irradiation, thermal decomposition, or hydrothermal/solvothermal treatment. The latter involves cutting down bulk carbonaceous materials (e.g., graphite powders, graphene, and black carbon) into nanosized materials through electrochemical oxidation, laser ablation, chemical oxidation, arc-discharge treatment, and ultrasonication.^16,28,110,112,123,145–148^ Like any other method, laser-based synthesis, such as LSPC, has its advantages and its fair share of disadvantages.^149–151^

**Figure 6:**
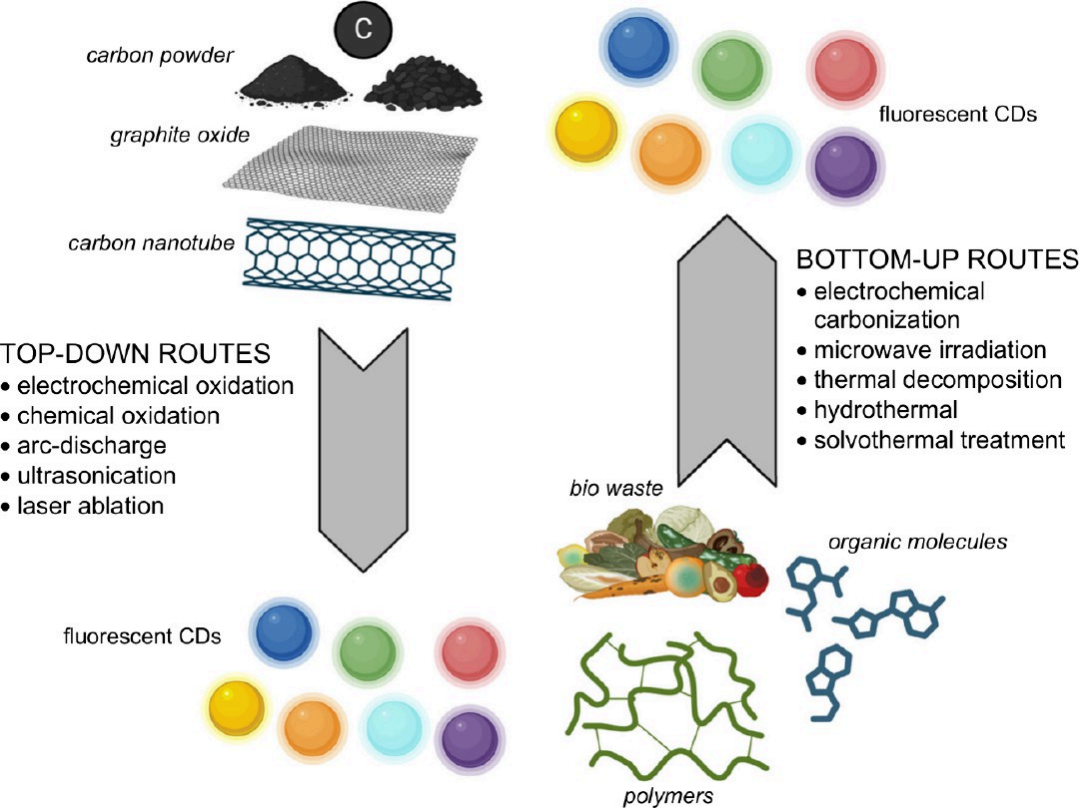
Top-down and bottom-up synthesis routes for CDs. Created in BioRender.com.

LSPC offers the advantage of being straightforward while enabling a rapid and easy implementation. Moreover, they provide a significant advantage over bottom-up methods in fabricating high-purity CDs, avoiding unnecessary toxic by-products and time-consuming purification processes.^53,54,73,149,152^ Apart from generating less chemical waste, this synthesis approach is considered green and eco-friendly because it operates without the need for any harsh chemicals, unlike chemical oxidation.^55,152,153^ In addition, Forzenetti et. al.^154^ demonstrated that the environmental sustainability of this method can also be extended to the possibility of recycling and reusing liquids, thereby minimizing the synthesis costs and environmental pollution. In terms of energy consumption and labor costs to produce NPs, they are comparable to, or in some cases lower than, those of conventional chemical synthesis method.^155,156^ For instance, under appropriate conditions such as the use of diffractive optical elements, Khairani et. al.^157^ showed that the energy usage, labor expenses, and ablation time in the production of NPs by multibeam PLAL can be reduced up to 50% in comparison to single-beam PLAL, making the method more cost-effective and economically sustainable. In addition to this, LSPC also does not require any complicated setup (e.g., vacuum chambers) and processing procedures since it only generally needs a target (carbon, in this case), the solvent of choice, and the laser to operate.^158^ Another advantage, as well, of this method is the capacity to control and produce the desired size, structure, and physicochemical properties of the CDs just by tuning different laser parameters such as fluence (energy per unit area), wavelength, pulse width, pulse frequency, and pulse energy, which remains a challenge for other CDs synthesis routes.^73,149^ The possibility of altering the surface functionality of CDs through modifying the solvent used leads to the capability of LSPC to *in situ* functionalization of the CDs.^70,159–162^ Overall, the key advantage of LSPC lies in its ability to fabricate a wide range of nanomaterials at a higher production rate, aiming for industrial-scale production and economic viability, while being an environmentally friendly method.^54,163^

Despite its numerous advantages, LSPC synthesis often gets overlooked due to certain drawbacks. One of the disadvantages commonly mentioned by the international scientific community is the availability, accessibility, and high cost of the laser system.^54,151^ Owing to technological advancements, the price of a standard laser system is continuously decreasing over time, and this disadvantage is slowly being overcome. For instance, the cost of a nanosecond laser is comparable to that of some equipment for making CDs in a wet chemistry laboratory. The affordability of highly efficient laser equipment opens more opportunities for laboratories to invest and participate in LSPC research, thus removing barriers to fostering collaborations among fields such as biology and material science. Today, the only issue this technique still experiencing is the low production rate, usually in the order of milligrams per hour (mg h^–1^). Another common disadvantage of this method is the laser-synthesized CDs usually possess low fluorescence quantum yield (QY) compared to bottom-up routes in a one-step synthesis process.^10,73,164,165^ The highest QY of CDs ever recorded is ∼97% in aqueous solution using a thermal method from the works of Liu et al.^166^ To our knowledge, the highest QY of laser-synthesized CDs is ∼47%, as reported by Narasimhan et al.^89^ Another issue this method faces is the hydrophobicity of common carbon precursors due to the existence of a strong covalent network of carbon in those precursors.^167^ They tend to be more dispersed in most organic solvents, which can be a significant problem in bio applications. Thus, to reduce the impact of these disadvantages, post-laser synthesis modifications are usually necessary to make CDs water-dispersible with enhanced fluorescence, which is more viable in bio-applications.^96,168^ Table 1 summarizes the advantages and disadvantages of LSPC (PLAL and PLFL) over other common synthesis methods (in terms of size and composition control, QY, pureness, green synthesis approach, scalability potential, and processability).

**Table 1:**
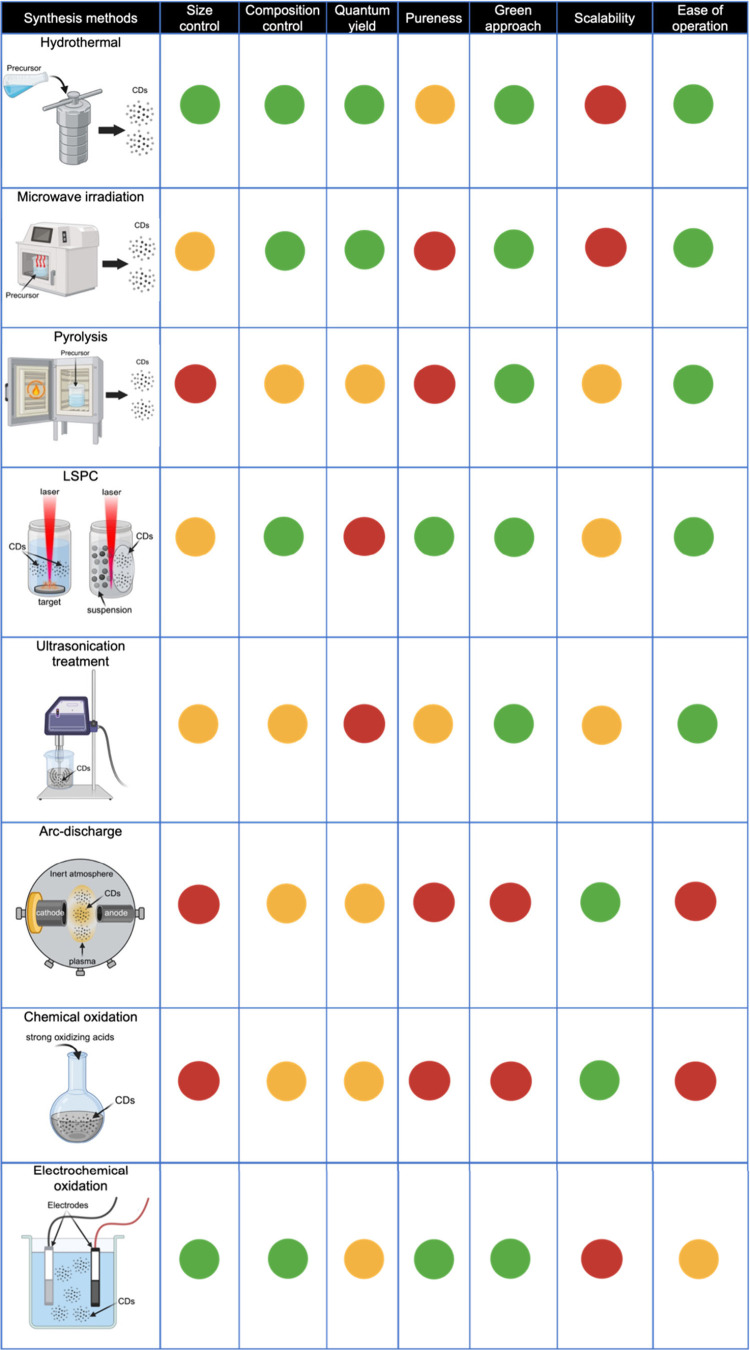
Advantages and Disadvantages of Various CDs Synthesis Routes^a^

a Green = excellent, yellow = fair/moderate, red = poor. Figures are created in BioRender.com.

## 2 Laser-Based Synthesis of CDs

There are four main techniques in LSPC-based synthesis of NPs depending on the starting material, implementation, and mechanism: (1) *laser ablation in liquids* (LAL), often known as “*laser ablation synthesis in solution*”, (2) *laser fragmentation in liquids* (LFL), (3) *laser melting in liquids* (LML), and (4) *laser reduction in liquids* (LRL).^53,54,159,169^ When a laser beam is directed to a bulk target submerged in a liquid to produce colloidal NPs, this method is known as LAL.^52,54,170^ LAL consists of three categories: pulsed laser ablation in liquids (PLAL), continuous laser ablation in liquids (CLAL), or reactive laser ablation in liquid (RLAL).^54,149,163,170–173^ As the name suggests, CLAL^174–176^ and PLAL^177,178^ differ in their laser sources if they emit a continuous beam of light (constant) or deliver high-energy pulses (pulsed). Furthermore, RLAL occurs if both the target and the liquid react together to form NPs due to laser excitation. On the other hand, LFL is another variation of laser-based synthesis where a laser beam is directed to micro powder suspensions or even colloidal NPs to reduce their size. In contrast to LFL, LML is a laser synthesis approach where a laser beam melts the precursor colloidal NPs to achieve either a size enhancement (nanoparticle size greater than the starting NPs) or a size modification (reshaping to different configurations, e.g., nanorods, nanowires, nano chains).^60^ If the liquid participates in NP formation during LFL or LML, this process is known as reactive-LFL or reactive-LML, respectively. LRL is another approach where the target is a solvent or a solution containing the compound of the desired NPs composition.^169^ The mentioned derivatives of LSPC showed that the technique is both a top-down NPs synthesis approach and a bottom-up approach. Apart from the mentioned laser-based synthesis derivatives, introducing defects and altering NPs properties without significantly changing the size of NPs is possible with a new laser methodology called *laser defect-engineering in liquid* (LDL).^53^ This clearly shows the versatility of the laser-based synthesis method in terms of NPs synthesis, their structural modification of NPs, and even altering NPs properties through a laser. In the following section, we will explore the mechanism of how NPs formed in general through PLAL and PLFL. However, some excellent reviews from Zhang et al.^54^ and Zeng et al.^170^ explain the different mechanisms of most LSPC-based syntheses in detail.

### 2.1 Fundamental Aspects of PLAL and PLFL Synthesis Approach

#### 2.1.1 Experimental Setup for PLAL and PLFL

The classic experimental setup for PLAL synthesis usually employed a vertically oriented focused laser beam that directly hit a fixed target at the bottom of the container, partially filled with liquid (Figure 7a).^54,73,97,149,158^ However, various established modifications of the PLAL experimental setup allow a more efficient NP synthesis than the classical setup. For example, a container containing a fixed target is placed at the top of an XYZ-translational stage^179,180^ or a rotational stage^181^ to avoid crate formation on the target (as shown in Figure 7a). Some researchers even employed ultrasonication bath^182^ and a uniform electric field^183,184^ (Figure 7b) to assist in the generation of the NPs. In the case of PLFL synthesis, the laser beam oriented vertically (can be focused or unfocused) directly hits the powder dispersion with a magnetic stirrer to avoid gravitational settling of powder and attain a maximum and uniform distribution of powder particles (Figure 7c).^54,73,149,163,170,172^ The laser beam for PLFL can be in horizontal^91,114^ and vertical orientation^185^ (Figure 7c). The vertical orientation can be advantageous if the laser beam is focused deep enough in the liquid to avoid evaporation and splashing of the powder dispersion. If, in any case, the laser beam causes the solvent to evaporate, a horizontal configuration is a good alternative due to the possibility of incorporating a lid or a cover. In addition, the latter configuration can also avoid splashing of the powder mixture. However, the drawback of this configuration is the energy loss of the laser beam since not all energy gets delivered to the solution.^73,149,173^ Alternative PLAL approaches also utilized a uniform external electric field^92^ or an ultrasonication bath^91,164^ rather than the magnetic stirrers to disperse the powders and to avoid contamination caused by stirrers at the same time. A liquid-flow-jet configuration (Figure 7d) has been proposed, where the powder dispersion is delivered by a motor pump and irradiated by the laser beam. As reported by Doñate-Buendía et. al.,^10^ this flow-jet strategy efficiently transformed 84% of the original carbon micro powder content to fluorescent CQDs compared to the conventional PLFL system, where it transformed only 64% of the original powder dispersion.

**Figure 7:**
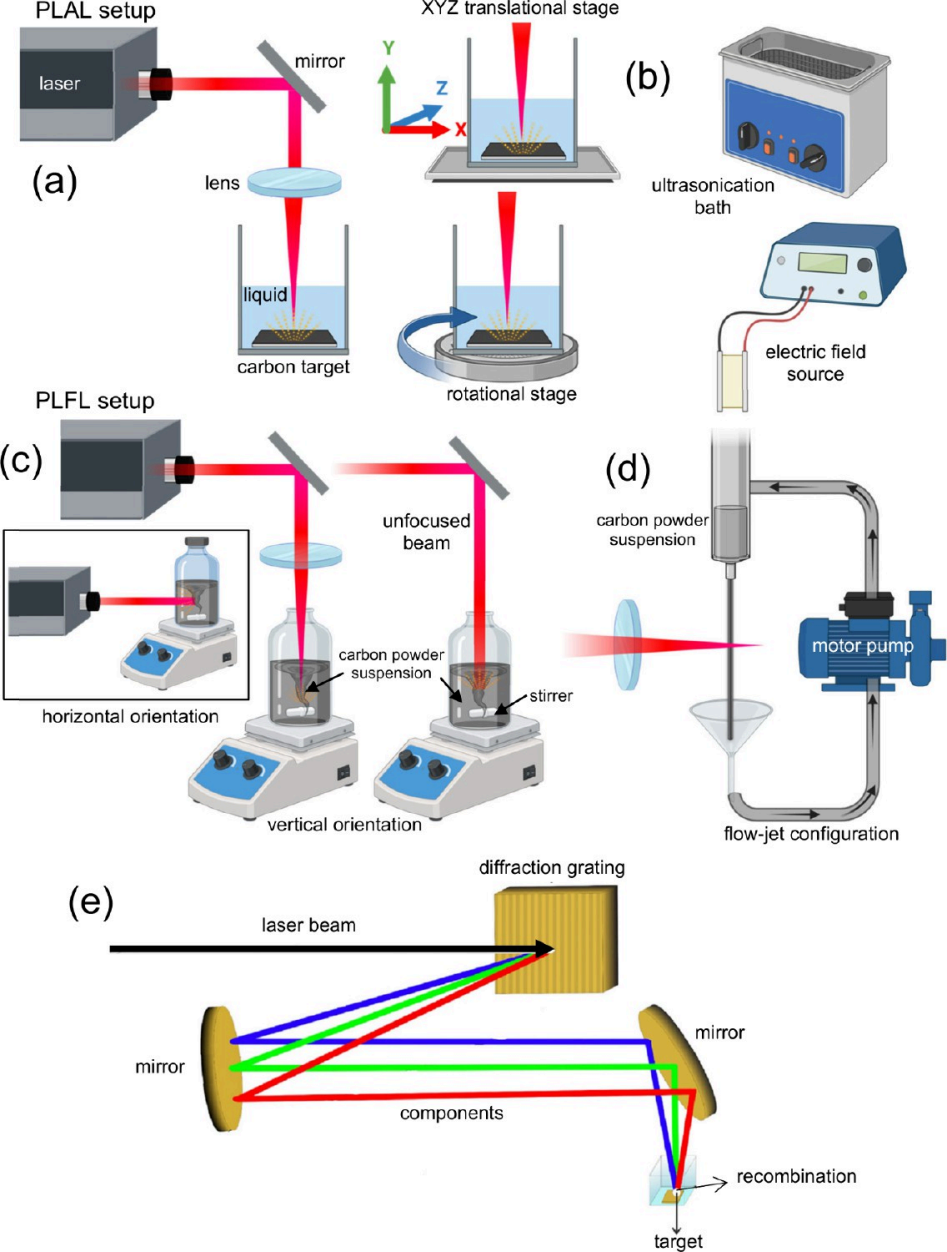
(a) Classic PLAL experimental setups together with the incorporation of translational, rotational stage, (b) ultrasonication bath and electric field, (c) PLFL experimental setups (horizontal and vertical beam orientation), and (d) flow-jet configuration technique for CDs synthesis (Created in BioRender.com). (e) SSTF technique for NPs synthesis (Reprinted with permission from ref 186. Copyright 2019 Optica Publishing Group).

As the laser-based synthesis of NPs has grown and reached different applications, researchers engineered and designed various modifications apart from the aforementioned setups in both PLAL and PLFL synthesis. These innovations mainly intend to maximize NPs productivity, improve NPs properties, and effectively deliver laser energy to the target. For example, Doñate-Buendía and coworkers^186^ developed a novel method to produce NPs through a technique they called *simultaneous spatial and temporal focusing* (SSTF) of laser pulses, where a laser beam passes through a diffraction grating, separates into different components, and recombines directly unto the target (Figure 7e). This method alleviates the energy loss through bypassing the previous interaction with the liquid compared to the classic ablation process for ultrashort lasers. With this technique, the energy delivered to the target increases by up to 70%, which results in an enhancement of the nanoparticle production rate by up to a factor of 2.4. Moreover, Cui et al.^187^ utilized a dual-beam pulsed laser ablation system to produce NPs other than a conventional single beam. The ablation efficiency of the dual beam significantly improved compared with the usual single-beam system. Additionally, Khairani et. al. used a diffractive optical element that generates multiple beams with foci directly hitting the target, increasing the production rate to a gram per hour (g h^–1^). The utilization of multiple beams reaches a production rate of 1.6 g h^–1^ for iron–nickel alloy NPs (production rate increase by a factor of 4) and 0.94 g h^–1^ for gold NPs (production rate increase by a factor of 3).^157^ In addition to the above-mentioned laser system modifications, further exploration of novel configurations such as the utilization of laser beam shaping techniques represents a promising approach to advancing the NPs production rate for industrial demands.

#### 2.1.2 NPs Formation through PLAL and PLFL

Laser ablation involves the removal of material based on the complex interaction process between a laser and a sample target. Its mechanisms are still under intensive investigation due to the dependence of the laser and target interaction process on several parameters such as laser pulse duration, laser energy, wavelength, properties of the target, and environmental composition.^188,189^ Based on pulse duration starting from nanosecond pulse and extending to femtosecond pulse regimes, this section provides general insight into the different mechanisms, neglecting other variables to simplify the laser ablation process.

The laser ablation process begins when a target of interest absorbs a high-power nanosecond laser beam. This process of PLAL is associated with the “*laser-matter interaction*” stage (Figure 8a).^53,54,73,97,149,159,190^ The ablation can occur if the laser fluence exceeds the material target’s ablation threshold, an intrinsic property for every material.^149,172^ During this stage, the absorbed laser energy excites electrons and transfers this energy to the lattice, which results in target heating. The increase in temperature eventually leads to the melting and vaporizing of the target material’s surface, and then plasma is generated.^152,170^ At the same time, the heat redistributed within the material from the melting zone and created a heat affected zone (HAZ). The ejected material is converted into a plasma plume near the ablated area, as the next stage of the PLAL process is known as the “*plasma phase*” (Figure 8b).^54,73,97,159,190^ This plasma plume, of high temperature and high pressure, is loaded with a very dense amount of ionized and atomized species. Part of the plasma recombines and is directly ejected into the liquid due to its high velocity, forming the primary NPs. In the case of the remaining plasma plume, the surrounding liquid absorbs energy as the plasma decays. It cools, vaporizing the surrounding fluid and creating a thin vapor layer around the plasma border.^152,173^ This provides a zone of active chemical interaction between the molecules from the surrounding liquid and the plasma plume, which leads to solvent decomposition. The breakdown of solvent molecules may be initiated by their interaction with the high-temperature plasma (known as “*thermal-induced liquid decomposition”*). Aside from the thermal decomposition of the liquid, high-energy photons can also be absorbed by the liquid molecules, leading to their dissociation and the formation of some radicals (e.g., ^•^OH, ^•^CH_3_, and O_2_^•–^ depending on the solvent composition). This is known as “*photon-induced liquid decomposition”*. The liquid molecules can also be dissociated through collision with high-energetic electrons from the plasma plume (known as “*electron-induced liquid decomposition*”).^54,169–171,191^

**Figure 8:**
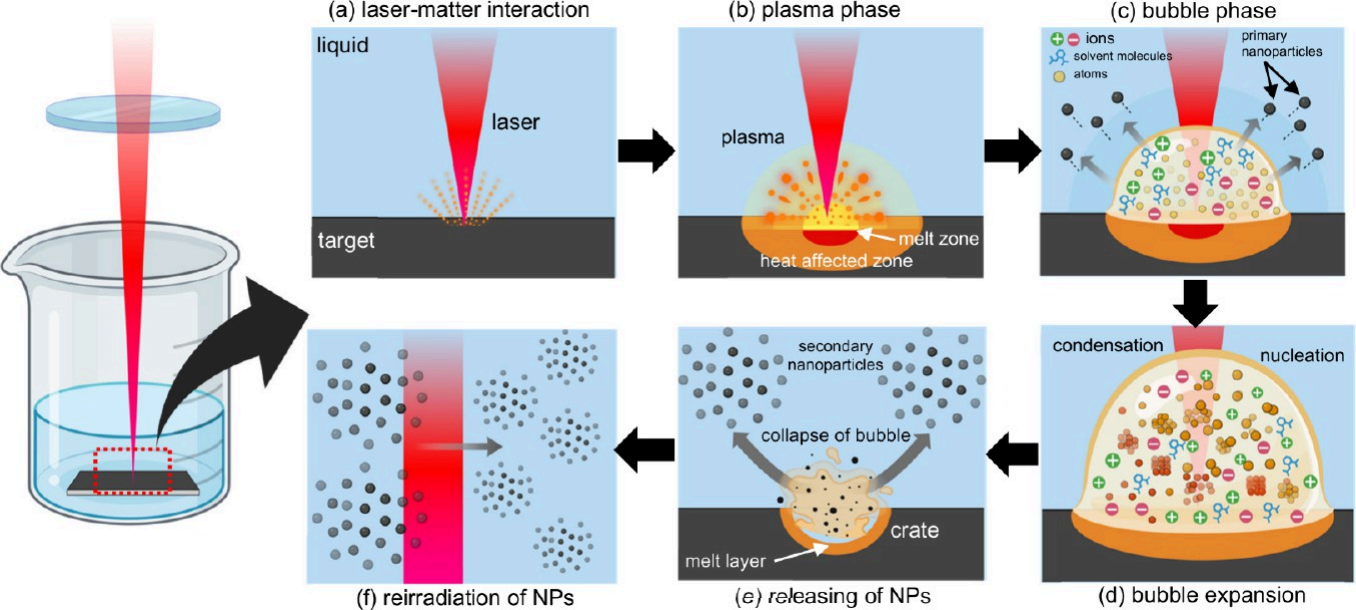
Stages of nanoparticles formation via PLAL technique: (a) laser-matter interaction stage, (b) plasma formation stage, (c) cavitation bubble formation stage (primary nanoparticle ejection), (d) expansion of cavitation bubble and secondary nanoparticles formation stage, (e) release of secondary nanoparticles stage to liquid, and (f) further reirradiation of formed nanoparticles. Created in BioRender.com.

The vapor subsequently expands to form a cavitation bubble to reach equilibrium with the surrounding liquid, where the bubble confines the dissociated components from the liquid, ablated ions, and atomic species from the material. This stage of the PLAL process is known as the “*bubble phase*” or “*gas phase*” (Figure 8c).^54,97,190^ While the cavitation bubble undergoes a fast expansion rate, the species inside it combine and react in the form of condensation and nucleation, forming another set of NPs (secondary NPs) (Figure 8d).^152^ In the final stage, the cavitation bubble experiences a series of expansion and shrinkage until it collapses and releases the secondary NPs onto the surrounding liquid (Figure 8e), leaving a considerable melt layer on the target.^73,152,159^ The released NPs (both primary and secondary) can encounter further size reduction because of their possible interaction with the laser beam, which leads to a laser fragmentation process (Figure 8f). In this case, the probability of laser fragmentation is greater unless the experimental design allows the released NPs to be removed shortly from the ablation setup through a vacuum, a motor pump or any means to avoid the re-irradiation.^149^ As shown above, the ablation process of long pulse duration, such as nanosecond (ns) lasers, greatly relies on heating, melting, and evaporation. Vaporization and melt expulsion (solid-melt–vapor transition) drive the removal of material. For this reason, this ablation mechanism is known as heating-melting-evaporation (HME), which dominates in both low and high laser fluence ns-laser regime.^59^

In ultrashort lasers such as femtosecond (fs) lasers, the absorption mechanism and ablation process differ from those of ns-laser. When laser energy absorbs in a target material, electron-to-lattice energy transfer time (*τ*_el_) and electron heat conduction time (*τ*_eh_) typically last in the order of ∼1 ps regardless of pulse duration (*t*_p_).^163,170,188,189^ Since *t*_p_ of a fs-laser pulse is shorter than both of these relaxation times (*τ*_el_ and *τ*_eh_), the laser energy absorbed rapidly by the material with no significant heating and thermal damage while avoiding leaving a noticeable melt layer on the ablation area. The absorbed energy then leads to the ionization of electrons and, subsequently, their thermalization on the target’s surface while leaving the ions below them (“cold ions”) initially at room temperature after the end of the laser pulse. The energy is then transferred from electron to lattice and ions, leading to electron-lattice heating and eventually vaporizing the target material, bypassing the melting phase (direct solid-vapor phase transition).^188,189^ The ionization of the target by fs-laser is made possible due to its high peak intensity (>10^13^ W/cm^2^) on a focused beam mode where the nonlinear absorption process becomes significant. Free electron generation via ionization mainly contributes to fs-laser plasma generation since thermal effects become negligible or non-existent in this regime (*t*_p_ ≫ *τ*_eh_). Multiphoton and electron impact ionizations are the two major ionization processes in free electron generation. Multiphoton ionization occurs when a bound electron becomes free after absorbing several photons such that the total absorbed energy is greater than its ionization potential and overcome the work function (minimum energy required to eject electrons) of the target material (Figure 9a). When this free electron re-absorbs laser energy via inverse Bremsstrahlung, it gains kinetic energy which, if sufficiently high enough, can free other bound electrons through collision.^163,170,188^ This process is called electron impact ionization (Figure 9b). After a series of collisions and reabsorption of laser energy, the population of free electrons can multiply, and thus, an electron avalanche occurs. The ablation of femtosecond lasers originating from ionization is called Coulomb explosion (CE). It is a nonthermal process where a laser beam ejects the electrons from the surface of the target material, leaving a highly charged surface behind.^54,59,170^ Due to the electron deficiency at the target surface, the internal charge repulsion becomes more vigorous. Eventually, it overcomes the intermolecular forces within, pushing the highly ionized atoms out of the bulk target and constituting the plasma (charge-separation effect). This mechanism is also commonly called “cold ablation” or “gentle ablation” owing to its ejection of ionized atoms and electrons without propagating excess heat in the lattice. This mechanism dominates at a low laser fluence regime near the ablation threshold.^54,172,192^ On the other hand, at very high laser fluence above the ablation threshold, the energy is so high that the material heats quickly, reaching a temperature above the vaporization point due to electron-lattice collisions. Then, there is a rapid thermal decomposition of the solid to vaporized species (ions and atoms), which leads to plasma formation. The ablation of fs-laser via thermalization is known as thermal vaporization (TV), which, in contrast with CE, is referred to as “strong ablation”.^189^ In addition, femtosecond laser ablation produces a plasma that consists of fast and highly charged ions (from CE) and slow and thermalized ions (from TV). The population of these ions depends on the laser fluence. The ns- and fs-laser ablation mechanisms presented here are explained based on the pulse duration, which determines the energy absorption mechanism, heating, and ablation process and extends to laser energy, which dictates to which extent these processes occur.^188,189^

**Figure 9:**
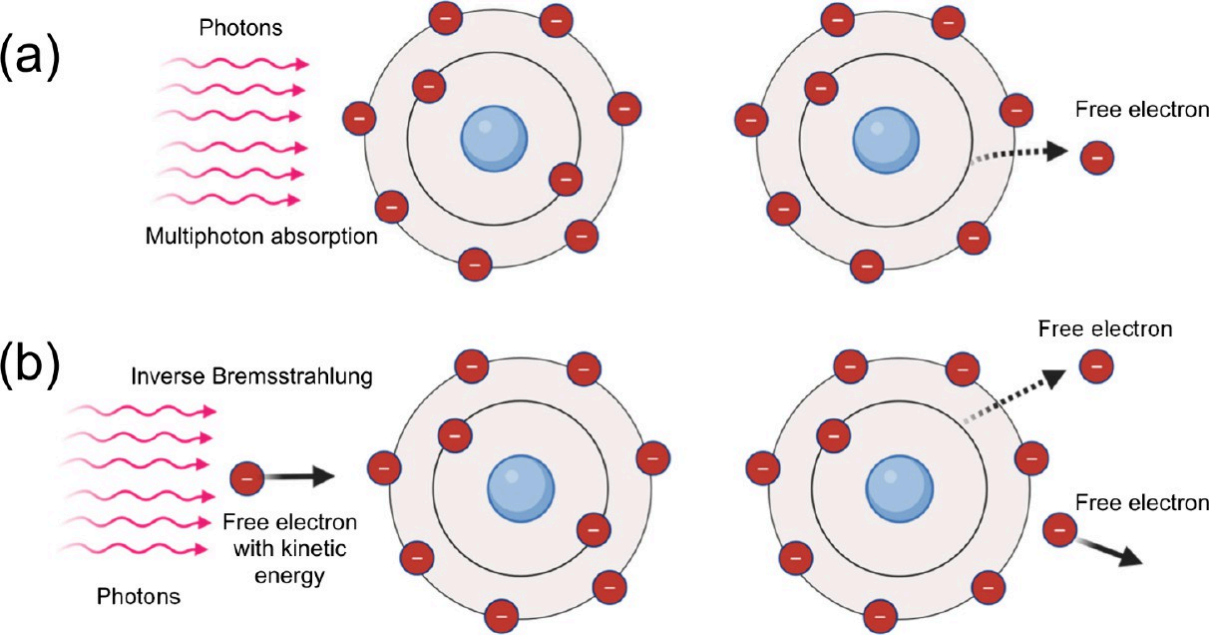
Nonlinear ionization processes: (a) Multiphoton ionization and (b) electron impact ionization (avalanche ionization). Created in BioRender.com

It is also essential to take note of the differences between the ns- and fs-laser ablations in terms of the ablation phases according to the characteristic times, *τ*_el_ and *τ*_eh_ defined above. When *t*_p_ ≫ *τ*_el_ and *τ*_eh_ (ns-laser regime), the laser pulse continually passes energy even after vaporization, plasma production, and cavitation bubble expansion (as shown in Figure 8b–8d). In other words, the absorption of laser energy and the material ablation occur simultaneously during the nanosecond pulse duration. For this reason, employing nanosecond laser can decrease the ablation efficiency due to different factors such as laser-plasma interaction, persistence of heat conduction losses within the target (greater HAZ and melting), and plasma shielding effects. The use of shorter nanosecond laser wavelengths reduces the magnitude of these factors. Employing fs-laser prevents all this from happening since the material removal, plasma formation, and cavitation bubble expansion occur at the end or after each pulse. Thus, minimal or negligible heat conduction losses (smaller HAZ and less melting) and no laser-plasma interaction.^188,189^

Although PLFL differs from PLAL in terms of its target material, the formation mechanism of NPs is quite similar. Instead of a bulk target, the PLFL utilizes a micro powder or colloidal suspension as its target.^54,73,163,170^ As the “laser-matter interaction” occurs between the suspension matrix and the laser beam, the target transfers heat to the liquid, forming a nanobubble similar to a cavitation bubble in PLAL. After, a plasma forms and fragmentation of micro powder occurs, followed by the recombination and condensation of the ionized species until they reach thermodynamic equilibrium, leading to NPs forming. Even though the fragmentation mechanism is still under debate, there are two established mechanisms similar to PLAL: thermal ablation and Coulomb explosion. Thermal ablation leads to the melting and/or vaporization of microparticles into atomized or ionized species. It is said to be dominant in ns-laser (Figure 10a). In contrast, the Coulomb explosion mechanism leads to the splitting and fragmenting of the larger particle into several smaller ones due to the large internal repulsion (Figure 10b).^54,59,170^ This fragmentation mechanism is said to dominate for the femtosecond laser of high laser fluence. Thus, the dominant mechanism over the other in the fragmentation process strongly depends on the pulse duration and laser intensity.^54,59,170^ Size reduction and selectivity can be achieved through PLFL with appropriate laser fluences and laser wavelengths to match the fragmentation threshold based on the starting particles while avoiding solvent evaporation. Uniform size distribution of NPs is possible through PLFL by controlling the different laser parameters. For instance, uniform size distribution is more pronounced in fs-laser than ns-laser.^53^

**Figure 10:**
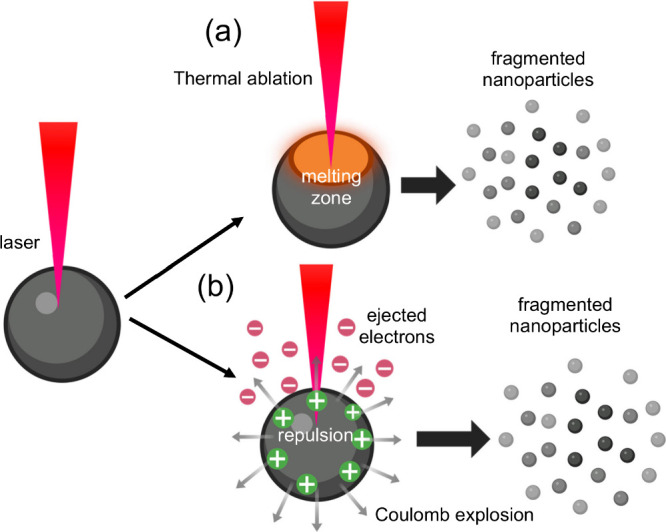
PLFL mechanism for nanoparticle synthesis: (a) thermal ablation and (b) Coulomb explosion mechanism. Created in BioRender.com.

### 2.2 Factors Influencing Laser-Synthesized CD Properties

A synthesis route alters CDs’ physicochemical, morphological, and structural properties by choosing different parameters, which depend on the synthesis approach used.^5,6,105,121,147^ In this case, the main components of PLAL or PLFL for CD formation are the laser source, liquid environment, and target. Regarding the laser source, the properties of CDs synthesized via PLAL or PLFL can be tuned or enhanced by changing the laser parameters, such as laser fluence, laser wavelength, pulse duration, or irradiation time. In addition, the desired surface functionality or the general chemical composition of CDs synthesized via these methods depend on the solvent used in the operation. The synthesis medium can vary from organic, inorganic, and ionic liquids to a mixture of different solvents, etc. Lastly, the target or the starting material for this method can significantly affect also the final properties of the CDs. At this point, we will explore the different parameters of PLAL and PLFL synthesis of CDs and how they can affect the final properties and functionality of CDs for their desired applications.

#### 2.2.1 Laser Parameters

##### Fluence

Laser fluence is the amount of energy (J) delivered to an area (cm^2^). Nguyen and co-workers^193^ examined the effect of laser fluence on CDs’ optical and structural properties. A Ti:Sapphire fs laser with a central wavelength of 800 nm, pulse duration of 150 fs, and repetition rate of 1 kHz was employed to synthesize CDs from carbon powder dispersion in polyethylene glycol (PEG) solution. They investigated the effect of laser fluence on CDs by focusing the carbon powder suspension at three different fluences: 150 J/cm^2^, 350 J/cm^2^, and 750 J/cm^2^. At a constant ablation time of 3 h, they discovered that the CDs’ mean size increases with fluence. The fluences 150 J/cm^2^, 350 J/cm^2^, and 750 J/cm^2^ produced CDs mean sizes of 1.5, 1.7, and 2.5 nm, respectively, as shown by their transmission electron microscopy (TEM) images (Figure 11a–11c). They attributed this trend of CD size reduction as the fluence increases to the cavitation bubble size and lifetime in which the increase of laser fluence also resulted in an increase in bubble lifetime. Consequently, the plasma inside the bubble nucleated for a longer time, increasing the CD’s size. Regarding their optical properties, CDs prepared at a fluence of 150 J/cm^2^ exhibit a maximum emission at 340 nm, while those prepared from 350 J/cm^2^ and 750 J/cm^2^ exhibit a red-shifted maximum emission at 410 and 480 nm, respectively. However, CDs prepared at 150 J/cm^2^ have a greater PL yield of 13.6% compared to the other two fluences: 350 J/cm^2^ has a PL yield of 6.2%, and 750 J/cm^2^ has a PL yield of 3.4%. Thus, CDs prepared from low laser fluence exhibit stronger PL emission.

**Figure 11:**
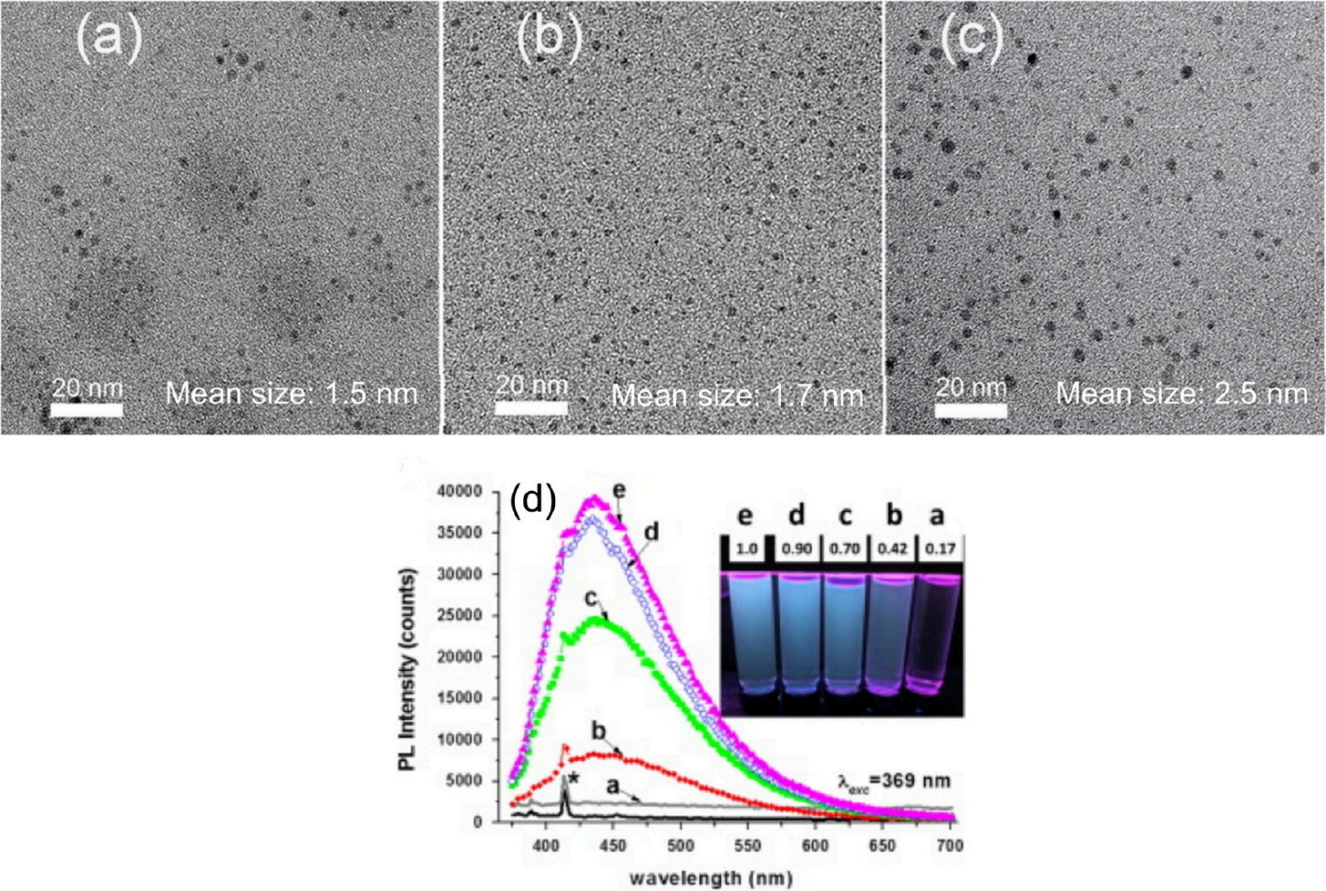
TEM images of CDs synthesized at different laser fluence: (a) 150 J/cm^2^, (b) 350 J/cm^2^, and (c) 750 J/cm^2^ (Reprinted with permission from ref 193. Copyright 2015 American Institute of Physics Publishing). (d) Fluorescence spectra of CDs synthesized at different fluences as indicated: (a) 0.17, (b) 0.42, (c) 0.70, (d) 0.90, and (e) 1.0 J/cm^2^, where the asterisk (*) represents the equipment lamp. Inset: Corresponding fluorescence of CDs at different fluences under UV lamp. (Reprinted with permission from ref 194. Copyright 2015 Elsevier Ltd.).

Reyes-Contreras et al.^194^ also investigated the effect of fluence, specifically on the fluorescence of CDs. To synthesize CDs, a graphite target was immersed in acetone and then ablated by using a Nd:YAG laser of 1064 nm laser wavelength and 15 Hz repetition rate while varying the laser fluence: (a) 0.17, (b) 0.42, (c) 0.70, (d) 0.90, and (e) 1.0 J/cm^2^. All CDs fabricated from these laser fluences exhibit a blue-green fluorescence of 440 nm emission wavelength. Interestingly, they discovered that CDs prepared at a higher fluence (1.0 J/cm^2^) have a strong emission under a UV lamp. In comparison, those at lower fluence (0.17 J/cm^2^) have a negligible or weak emission, as shown in Figure 11d. This is also in agreement with their measured fluorescence spectra, as shown in Figure 11d. This study shows that there is a strong correlation between the laser fluence used and the resulting fluorescence of the CDs.

##### Wavelength

Reyes and co-workers^195^ investigated the effect of ns-laser pulses of different wavelengths on the properties of the CDs. They employed a solid-state laser (Nd:YAG) to ablate a solid carbon target immersed in pure acetone at different wavelengths (fundamental emission of 1064 nm, second harmonic emission of 532 nm, and third harmonic emission of 355 nm) with a constant fluence of 12.5 ± 0.5 J/cm^2^. They observed increased agglomeration and large clusters formed from 355 to 1064 nm, as shown in their TEM images (Figure 12a–12c). The ablation efficiency of 355 nm is lower than those of the other two wavelengths due to the lower optical penetration of 355 nm in the material and the unavoidable absorbance of acetone at this wavelength. Additionally, they found that the fluorescence of synthesized CDs was higher when a 355 nm laser was used (at a constant irradiation time of 150 s) compared with the other wavelengths at the optimal excitation energy of 3.54 eV (350 nm). However, at higher irradiation time (900 s), the fluorescence of CDs synthesized using 532 nm enhanced significantly compared to the other wavelengths (Figure 12d).

**Figure 12:**
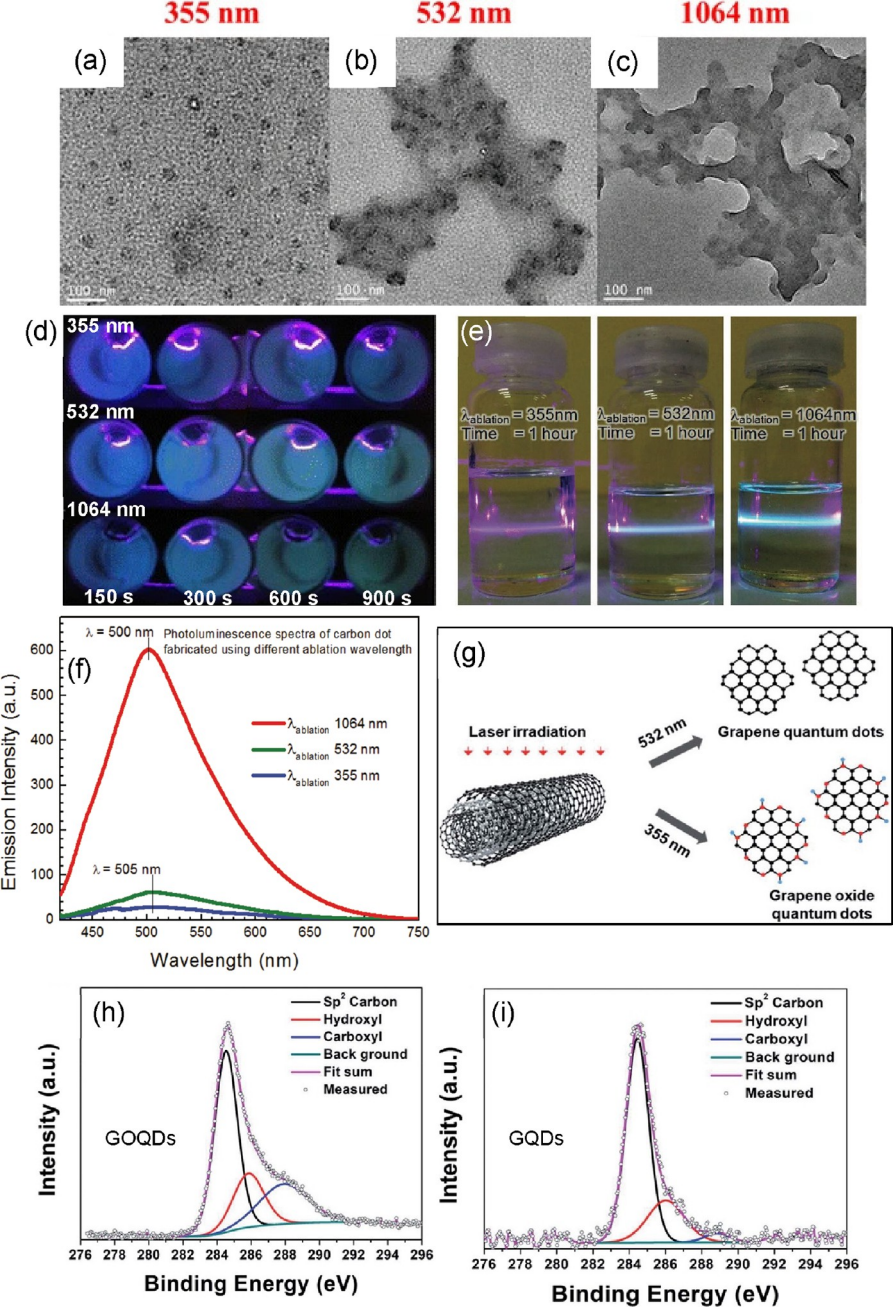
TEM images of CDs synthesized at different laser wavelengths: (a) 355 nm, (b) 532 nm, and (c) 1064 nm. (d) Fluorescence emission of CDs synthesized at different laser wavelengths with varying irradiation time under UV lamp (Reprinted with permission from ref 195. Copyright 2016 Springer Nature). (e) Fluorescence emission of CDs synthesized at different wavelengths under violet laser pointer and their (f) corresponding fluorescence spectra (Reprinted with permission from ref 196. Copyright 2017 American Institute of Physics Publishing). (g) Schematic representation of the type of GQDs produced from MWCNT ablated at different wavelengths and the XPS spectra of (h) GOQDs and (i) GQDs, respectively (Reprinted with permission from ref 197. Copyright 2019 Royal Society of Chemistry).

However, Isnaeni et al.^196^ discovered that the 1064 nm laser produces a high bright-blue fluorescence emission of CDs instead of other wavelengths (355 and 532 nm), as shown in their fluorescence appearance when excited by a violet laser pointer (Figure 12e). They synthesized their CDs using the PLFL technique, where they started with carbon powder dispersion in toluene and ablated at different wavelengths but with constant energy (60 mJ) and constant ablation time of 1 h. They also detected a slight shift of maximum emission wavelength from 505 nm (CDs synthesized by 355 and 532 nm lasers) to 500 nm (in the case of 1064 nm lasers) as shown by their fluorescence spectra (Figure 12f). They attributed this emission shift from the effective size reduction of CDs of 1064 nm-laser following the size effect mechanism of CDs fluorescence.

Another study by Kang et al.^197^ showed that the PLFL process can fabricate GQDs or GOQDs (graphene oxide quantum dots) solely by modulation of laser wavelength. Structurally, GQDs and GOQDs differed by increasing the number of oxygen-rich functional groups (e.g., −OH or −COOH) on their edges/surfaces. To fabricate these QDs, they used MWCNTs (multiwalled carbon nanotubes) as carbon source dispersed in high-purity ethanol using a horizontal pulsed laser of wavelength 355 and 532 nm, repetition rate of 10 Hz and ablation energy of 50 mJ. They demonstrated that PLFL using a 532 nm laser source can produce GQDs. In contrast, a 355 nm laser can make GOQDs (Figure 12g). The abrupt increase of hydroxyl and carboxyl groups on the GOQDs surface rather than on the GQDs surface is the result of the effective photothermal decomposition of ethanol by the high-photon energy of the 355 nm laser. The researchers utilized the X-ray photoelectron spectroscopy (XPS) technique to investigate and quantify their surface functionality. They found out that hydroxyl and carboxyl comprised 44% of the GOQDs XPS spectra (Figure 12h) while only 13% of the GQDs XPS spectra (Figure 12i). Additionally, the reaction of the laser beam with ethanol induces an *in-situ* surface functionalization of the QDs thus, this method classifies as RLFL.^171^

##### Pulse Duration

Pulse duration (or pulse width) refers to the temporal width of the laser pulse. Recently, Shatov et al.^198^ studied the effect of these laser pulse durations on the bottom-up laser synthesis of carbon dots from pure toluene. They used a 1033 nm laser with a constant pulse energy of 2 mJ and varying pulse durations of 0.25, 1, 3, and 10 ps. The absorption of a 1033 nm laser wavelength by toluene molecules leads to ionizing and dissociating the toluene structure. As a result, the plasma, composed of ionized and reactive species, subsequently reacts to form fluorescent CDs. In this case, they found that there is a correlation between the final chemical composition of the CDs and pulse duration. With the increase of pulse duration (10 ps), they discovered a significant number of graphite-like domains and sp^2^ carbon atoms, which are associated with the CDs structure, formed from the fused aromatic rings of toluene. Moreover, they found more isolated aromatic rings using a pulse duration of 0.25 ps (Figure 13a). Regarding their fluorescence properties, they observed that all synthesized CDs at different pulse durations exhibit blue fluorescence intensity with an excitation wavelength of 360 nm. However, the researchers found out that the 10 ps CDs have a greater fluorescence intensity with a relatively higher PL yield of 5.24% compared to other pulse durations: 0.25, 1, and 3 ps have a PL yield of 2.88%, 2.6%, and 5.07%, respectively. Furthermore, Batista et al.^199^ also demonstrated the effect of the pulse duration on the properties of bottom-up laser synthesis of CDs using various organic solvents (acetone, ethanol, *n*-hexane) as their carbon source. They investigated two pulse durations (30 fs and 4 ps) and found that the longer pulse duration (4 ps) can effectively form CDs of higher PL yield compared to 30 fs at the same ablation time of 1 h (Figure 13b). Hu et al.^85^ further demonstrated the effect of pulse width on the size and fluorescence of CQDs synthesized via PLFL using graphite flakes dispersed in a PEG solution using three laser pulse widths of 0.3, 0.9, and 1.5 ms. They found that as the laser pulse width increases, the CQDs size also increases; that is, 0.3 0.9, and 1.5 ms produced 3.2 8.1, and 13.4 nm CQDs size, respectively. They concluded that the laser pulse width could affect the nucleation and NPs growth during the PLFL process, thereby controlling their size distribution. On the other hand, they also found that the synthesized CQDs exhibit size-dependent photoluminescence behavior, in which the fluorescence of 13.4 nm-CQDs was red-shifted for the 3.2 nm-CQDs (Figure 13c). Furthermore, the PL yield of 3.2 nm-CQDs (12.2%) is higher compared to the other two: 8.1 nm-CQDs have a 6.2% PL yield, while 13.4 nm-CQDs have a 1.2% PL yield.

**Figure 13:**
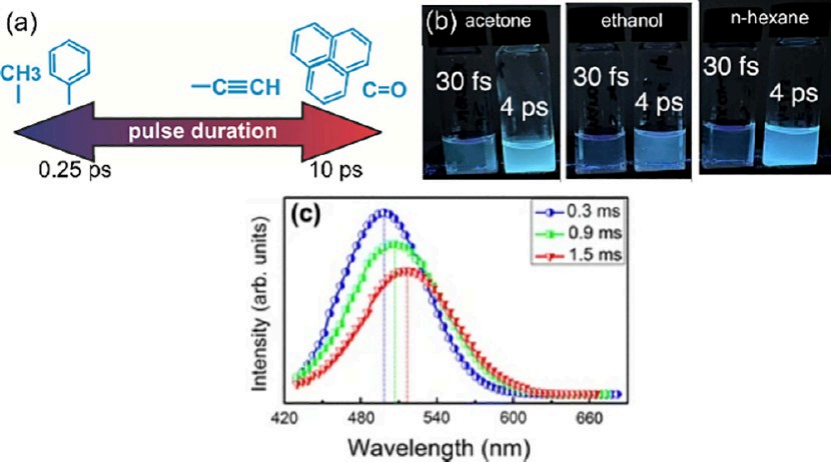
(a) Schematic representation of the product yield at different pulse durations from pure toluene molecules (Reprinted with permission from ref 198. Copyright 2023 Peter the Great St. Petersburg Polytechnic University); (b) Fluorescence emission of CDs synthesized directly from different organic solvents at two pulse durations (30 fs and 4 ps) under 365 nm illumination (Reprinted with permission from ref 199. Copyright 2023 American Chemical Society); (c) Fluorescence spectra of laser-synthesized CDs at different laser pulse duration (Reprinted with permission from ref 85. Copyright 2011 Springer Nature).

##### Irradiation Time

Russo et al.^200^ demonstrated a femtosecond laser fragmentation of graphene oxide dispersion in water to fabricate GQDs. At a constant repetition rate of 1 kHz and a pulse duration of 35 fs, they revealed a relationship between the irradiation time and the final nanostructure and quality of GQDs. By maintaining a fluence of 47.7 J/cm^2^ (laser power of 2.4 W), the researchers prepared four colloidal suspensions of GQDs with irradiation times of 5, 15, 30, and 60 min. Their TEM images show that the 5 min process (Figure 14a) does not exhibit any substantial modifications to graphene oxide sheets. In contrast, 15 min (Figure 14b) showed already the formation of several GQDs with a mean size of 2.3 nm. As the irradiation time increases to 30 min (Figure 14c), the average size increases to 3.0 nm and stays the same size even at 60 min. However, the longer irradiation time of 60 mins displayed other nanostructures made of an assembly of graphene sheets, where the GQDs were embedded.

**Figure 14:**
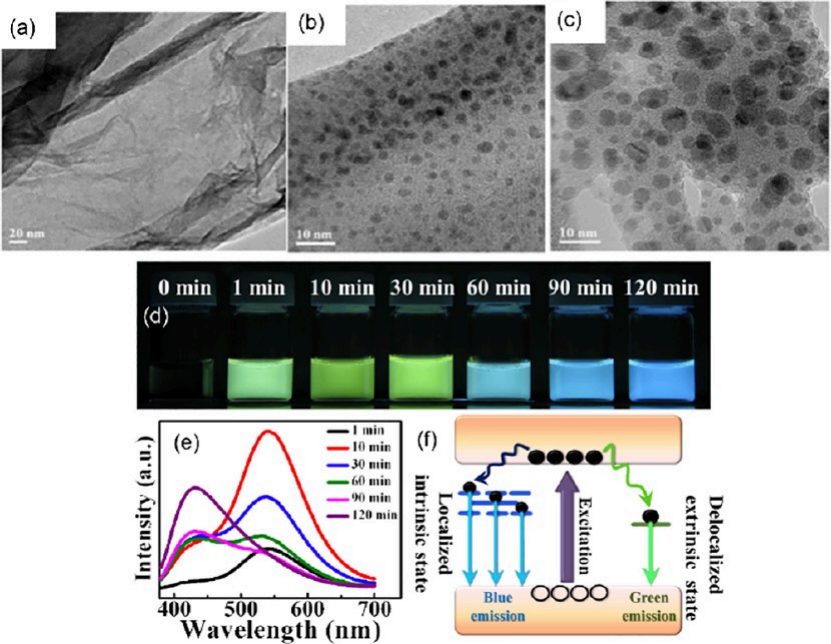
TEM images of laser modification of graphene oxide dispersion in water at different irradiation times: (a) 5 min, (b) 15 min, and (c) 30 min. (Reprinted with permission from ref 200. Copyright 2016 Royal Society of Chemistry). (d) Fluorescence emission of laser-synthesized GQDs at different irradiation times as indicated and (e) their corresponding fluorescence spectra. (f) Proposed mechanism of the origin of blue and green emission (Reprinted with permission from ref 87. Copyright 2016 Royal Society of Chemistry).

In contrast with Russo et al.’s investigation of the effect of irradiation time on GQD structure, Santiago et al.^87^ studied the effect of irradiation time exclusively on the fluorescence of GQDs synthesized from carboxyl functionalized MWCNTs. They used laser fragmentation to synthesize GQDs using a 415 nm-laser source with a frequency of 10 Hz and energy of 48 mJ but with varying irradiation times between 0 and 120 min. They determined that when the irradiation time increases from 1 to 120 min, the fluorescence of GQDs blue-shifted from their initial green emission dominance at 1 min, as shown in Figure 14d under a UV lamp. They found that the fluorescence spectra of all of the synthesized GQDs at different times exhibit a mix of two fluorescence peaks at 430 nm (blue) and 540 nm (green), as shown (Figure 14e). They observed that the blue emission dominates at longer times whereas, at a relatively shorter irradiation time, the green emission dominates. They hypothesized that the oxidation laser treatment occurs at longer irradiation times, resulting in a structural change of GQDs and attachment of more oxygen-functional groups (e.g., epoxy and carboxyl groups) on the GQDs surface. Furthermore, they proposed a mechanism for these two simultaneous emissions by GQDs in which the excited electrons have two relaxation pathways: one is the direct relaxation of electrons to the localized intrinsic states (sp^2^ nanodomains/carbon core) causing the blue emission while some electrons can be trapped on the extrinsic surface state causing the green emission (Figure 14f).

#### 2.2.2 Solvent Effects

Carefully selecting the solvent is essential in laser synthesis to achieve the desired quality of CDs. Due to their inevitable participation during laser irradiation, the chosen solvents can significantly affect CDs’ general characteristics and structure. Hence, this could lead to and contribute to the formation of defects, incorporation of dopant atoms, attachment of various surface states/traps, and general chemical composition of CDs, which might hinder or boost the performance of the CDs. Tarasenka and co-workers^201^ studied the effect of various liquids such as H_2_O, ethanol, and 0.008 M aqueous diethylenetriaminepentaacetic (DTPA) acid solution on the properties of CDs. Using an Nd:YAG laser of 532 nm-wavelength with a fluence of 660 mJ/cm^2^, they fabricated CDs from a bulk graphite target via PLAL. They observed that the synthesis performed in water led to amorphous CDs, while in DTPA solution and ethanol led to highly crystalline CDs (Figure 15a–15c). They further investigated and examined the core structure of CDs in DTPA and ethanol through high-resolution TEM (HR-TEM). Surprisingly, they observed that CDs in DTPA and ethanol possess a hexagonal diamond phase and an orthorhombic carbon phase, respectively. Additionally, the intrinsic luminescence of CDs was higher and red-shifted from a 400 nm blue emission of CDs in H_2_O to a green emission of 500 nm in DTPA. They explained that the shift arises from the formation of emissive sites established by nitrogen defects and surface groups from the addition of DTPA.

**Figure 15:**
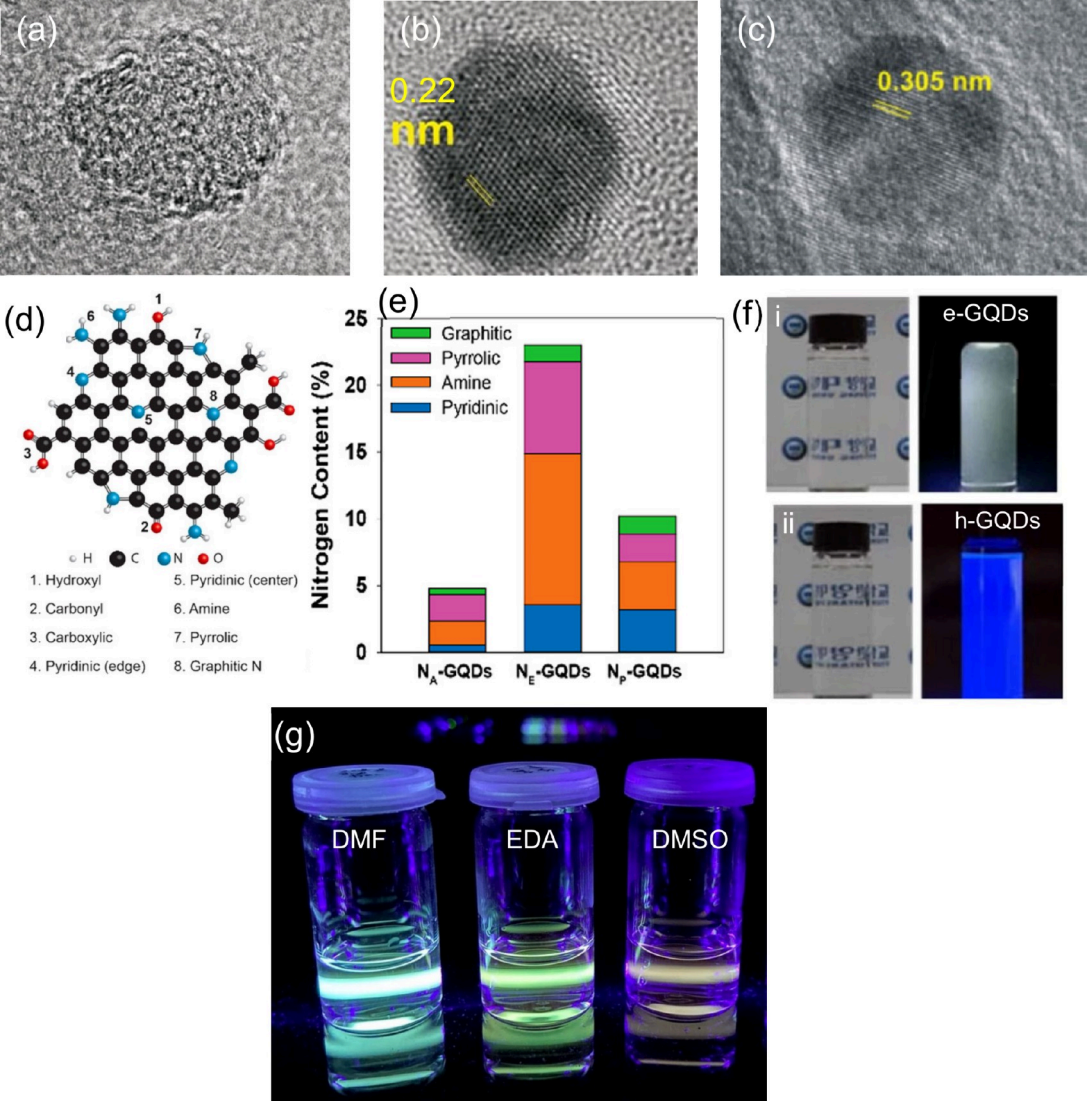
HR-TEM images of CDs synthesized in different liquid environments: (a) water, (b) diethylenetriaminepentaacetic (DTPA) aqueous solution, and (c) ethanol. Scale bars are 2 nm (Reprinted with permission from ref 201. Copyright 2017 John Wiley & Sons, Inc.). (d) Proposed structure of GQDs synthesized at different nitrogen-containing solvents. (e) Content of different nitrogen configurations in GQDs synthesized in ammonia (N_A_-GQDs), ethylenediamine (N_E_-GQDs), and pyridine (N_P_-GQDs) (Reprinted with permission from ref 202. Copyright 2019 American Chemical Society). (f) GQDs synthesized in (i) ethanol (e-GQDs) and (ii) hexane (h-GQDs) under ambient light (left) and 360 nm lamp (right). (Reprinted with permission from ref 203. Copyright 2016 Springer Nature.) (g) Fluorescence of CQDs synthesized in DMF, EDA, and DMSO using a laser pointer of 405 nm wavelength.

The exploration of solvent effect on the overall composition of CDs was continued further by the works of Calabro et al.^202^ They applied four solvents, namely, water, 2.5 M solutions of ammonia, ethylenediamine, and pyridine, to synthesize GQDs via PLAL from the pelleted carbon nanoonions using a 532 nm pulsed nanosecond laser. They named those synthesized GQDs prepared in water, ammonia, ethylenediamine, and pyridine as Ox-GQDs, N_A_-GQDs, N_E_-GQDs, and N_P_-GQDs, respectively. Then, they investigated the total nitrogen content and the distribution of functional groups on the surface or within the core structures of these four samples. They discovered that the solvent molecules dissociate into different reactive moieties and are then chemically bonded to the resultant GQDs during ablation rather than adsorbed physically on GQDs. In terms of nitrogen-containing solvents, the doping of nitrogen moieties leads to different configurations such as pyridinic (edge), pyridinic (center), amine, pyrrolic, and graphitic N as shown in (Figure 15d). Using high-resolution N 1s XPS spectra of the N-GQDs samples, they quantified the relative percentage of these nitrogen configurations on GQDs and found that N_E_-GQDs has a relatively higher amount of amine and pyrrolic N compared to N_A_-GQDs and N_P_-GQDs (Figure 15e). These N configurations directly influenced the emission of N-GQDs where N_A_-GQDs (amine N-dominated) had a red-shifted emission of 465 nm relative to N_P_-GQDs (pyridinic N-dominated), which had a blue-shifted emission of 415 nm. Moreover, the PL yield of Ox-GQDs is 0.81%, which is comparatively lower than the N-GQDs samples: N_A_-, N_E_-, and N_P_-GQDs had PL yields of 3.8%, 8.6%, and 4.6%, respectively. From this, they concluded that N doping can enhance GQD fluorescence emission. Among the N-GQDs samples, N_E_-GQDs had the highest PL yield due to their highest nitrogen content. In this study, they concluded that the ratios of different N-functional groups tune the PL properties of GQDs.

Kang and co-workers^203^ demonstrate another case of the impact of organic solvents on the laser-synthesized GQDs. Their experiment employed two organic solvents (ethanol and *n*-hexane) for the 355 nm-laser fragmentation of MWCNTs to produce GQDs. The laser exfoliated the outer walls of MWCNTs to fabricate GQDs in hexane (h-GQDs) and oxidized-GQDs in ethanol (e-GOQDs), both possessing a spherical morphology and a uniform size distribution between 1-5 nm. The amount of oxygen-related functional (hydroxyl and carboxyl) groups from e-GOQDs is higher than h-GQDs due to ethanol molecules’ photodissociation. Consequently, e-GOQDs exhibited a broader emission peak centered at 450 nm due to those functional groups. Furthermore, the PL yield of h-GQDs is 12%, which is higher than the PL yield of e-GOQDs (0.8%). These findings agree well with the fluorescence of these samples at 365 nm (Figure 15f), in which h-GQDs emit an intense deep blue emission. Overall, this study provides evidence that the laser synthesis of either GQDs or GOQDs can be achieved by simply changing the solvents. Additionally, Cortes et al. also showed that by changing the liquid environment of laser fragmentation synthesis of CQDs from carbon black powder, the fluorescence emission significantly changes where CQDs synthesized in dimethylformamide (DMF), in ethylenediamine (EDA) and dimethyl sulfoxide (DMSO) emit blue-green, yellow-green, and yellow fluorescence, respectively, using a laser pointer of 405 nm wavelength (Figure 15g).

Other than the organic solvents, Castro et al.^204^ redirected their work on PLFL synthesis of CQDs, focusing on ionic liquids (ILs). They performed laser fragmentation to synthesize CQDs by using a 1064 nm Nd:YAG laser to irradiate a graphite powder suspension in various ILs namely, 1-n-butyl-3-methylimidazolium tetrafluoroborate (abbreviated as BMI·BF_4_), 1-n-butyl-3-methylimidazolium bis­(trifluoromethanesulfonyl)­imide (abbreviated as BMI·NTf_2_), and 1-n-octyl-3-methylimidazolium bis­(trifluorome-thanesulfonyl)­imide (abbreviated as OMI·NTf_2_). They believed that utilizing these ILs could provide an excellent stabilization layer on CQDs and fluorescence enhancement of CQDs. By changing the anionic and cationic nature of ILs, the measured PL yield increases from BMI·BF_4_ (5%) < BMI·NTf_2_ (19%) < OMI·NTf_2_ (20%). Apart from that, the prepared CQDs in BMI·BF_4_, BMI·NTf_2_, and OMI·NTf_2_ have an average size of 2.9, 3.0, and 1.5 nm, respectively. Thus, changing ILs could also influence the final morphology of CQDs.

#### 2.2.3 Carbon Precursors

Besides the laser parameters and liquid medium, the target material also plays a vital role in determining the type of CD nanostructure formed after laser synthesis. Numerous reports have been regarding the PLAL and PLFL synthesis of CDs from various carbon sources, such as graphite flakes, carbon black powder, etc. However, this review will classify and generalize the carbon sources used in the laser synthesis approach, such as ***carbon allotropes***, *biomass*, and ***small organic molecules***.

***Carbon allotropes*** such as graphite flakes, graphite powder, carbon black powder, charcoal, carbon nanotubes, and diamonds are typical targets for PLAL and PLFL synthesis. For this reason, classic PLAL and PLFL synthesis are known as top-down CD synthesis approaches. Yogesh et al.^168^ studied the laser ablation of charcoal powder in ethanol to synthesize CDs using ns-PLFL at 1064 nm laser wavelength. They employed a post-laser modification process of the CDs obtained in ethanol to convert them into water-dispersible CDs (W-CDs) through a simple addition of water in the prior CDs solution. They found that W-CDs, with an average size of 21 nm (Figure 16a), were amorphous. Similar to the behavior of most CDs, W-CDs exhibit an excitation-wavelength dependent fluorescence with a maximum emission at 445 nm (Figure 16b). Moreover, Hu et. al.^205^ studied the different CDs structures formed from two powder targets in absolute ethyl alcohol: graphite flakes and carbon black. They employed a millisecond pulsed Nd:YAG laser of wavelength 1064 nm and a frequency of 20 Hz to obtain a colloidal suspension from the targets mentioned earlier. Although CDs from both targets had a very narrow size distribution with an average size of 3 nm (Figure 16c–16d), they differed by their crystallinity, where CDs from graphite flakes showed outstanding crystal features while those from carbon black exhibited several crystal defects on their core structure (Figure 16c–16d insets). To better understand the structure of the prepared CDs, the researchers employed Raman spectroscopy to distinguish structural disorders and hybridized carbon in CDs. Both samples possessed two Raman peaks centered at 1327 cm^–1^ (represents sp^3^ bonding and disorder) and 1582 cm^–1^ (represents sp^2^ bonding and graphitic phase) (Figure 16e). However, CDs from carbon black showed higher intensities of these peaks in comparison to CDs from graphite flakes. The researchers concluded that CDs produced from carbon black possessed more defects and a higher amount of sp^2^-hybridized carbon. Regarding their fluorescence, both CD samples possessed a strong emission around 490 and 510 nm. However, the PL yield of those from carbon black was 9.5%, which is lower compared to those from graphite flakes, which is 16.2% using 416 nm as the excitation wavelength. Furthermore, Dudek and colleagues^206^ also investigated the different CD characteristics in terms of their size and phase structures through 1064 nm laser ablation of two types of carbon targets submerged in water: graphite and polycrystalline diamond targets. They found that the CDs formed from graphite targets have a fullerene-like phase structure with higher disorder content, while CDs from diamond targets are a mixture of sp^2^/sp^3^ carbonized NPs.

**Figure 16:**
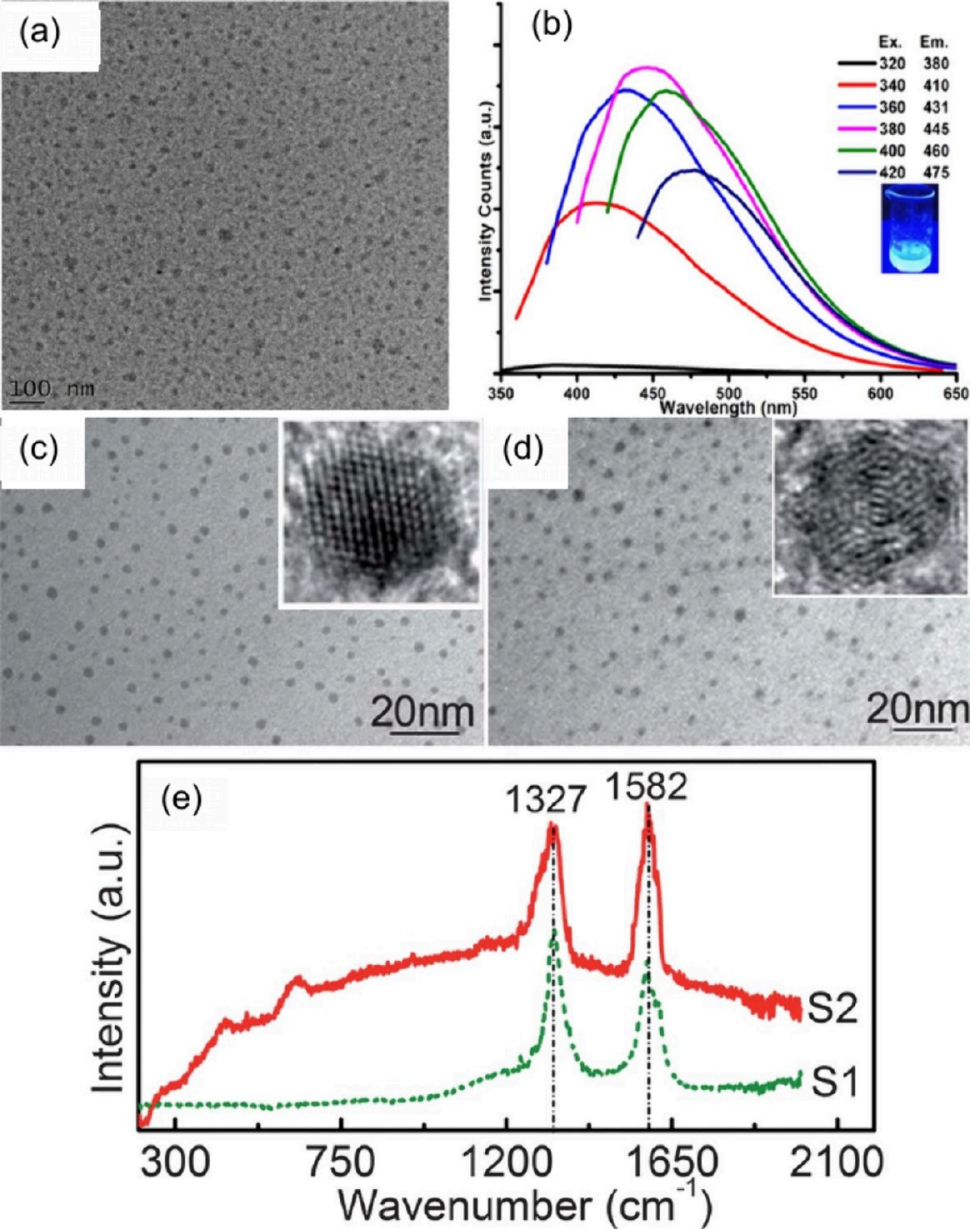
(a) TEM image of CDs synthesized from charcoal powder and their (b) corresponding fluorescence spectra at different excitation wavelengths (Reprinted with permission from ref 168. Copyright 2021 Elsevier Ltd.). TEM images of CDs from (c) graphite flakes and (d) carbon black powder. Inset: HR-TEM of CDs; (e) Raman spectra of CDs from graphite flakes (S1) and from carbon black powder (S2) (Reprinted with permission from ref 205. Copyright 2012 Royal Society of Chemistry).

***Biomass*** has been actively emerging as a carbon source for the laser synthesis of CDs due to its low cost and environmental friendliness. PLAL and PLFL can also be labeled as bottom-up synthesis approaches, as mentioned before. Jumardin et al.^207^ synthesized CDs from tea dispersed in toluene via PLFL by utilizing the 1064 nm laser wavelength of a Nd:YAG laser. The prevalent size of CDs from tea is around 6 nm, with a strong fluorescence at 496 nm. Additionally, Yogesh et al.^208^ utilized *Bougainvillea alba* flower extract to synthesize CDs via Nd:YAG ns-laser fragmentation synthesis in water (Figure 17a). The obtained CDs from the extract were polydisperse, with a mean size of 25 nm. The researchers found that the prepared CDs showed a presence of both amorphous and crystalline nature with a lattice spacing of 0.27 nm as shown by their HR-TEM (Figure 17b). The prepared CDs showed a common excitation-wavelength dependent behavior with the highest emission at 485 nm. Another case of laser-synthesized CDs from natural products was performed by Enriquez-Sanchez et al.,^58^ where they used waste coffee grounds as their carbon precursor. They first carbonized the coffee waste using a tubular oven. Then, they performed laser fragmentation of the obtained powder in four different solvents (acetone, toluene, methanol, and isopropyl alcohol) using an Nd:YAG laser of 1064 nm wavelength. They found that the synthesized CDs from coffee waste in four different solvents had the same broad photoluminescence emission centered at 430 nm. Still, CDs in toluene possessed a stronger emission than other solvents. Interestingly, they also discovered that the CDs in acetone, with an average size of 45 nm, were spherical and embedded in a carbon nanosheet matrix.

**Figure 17:**
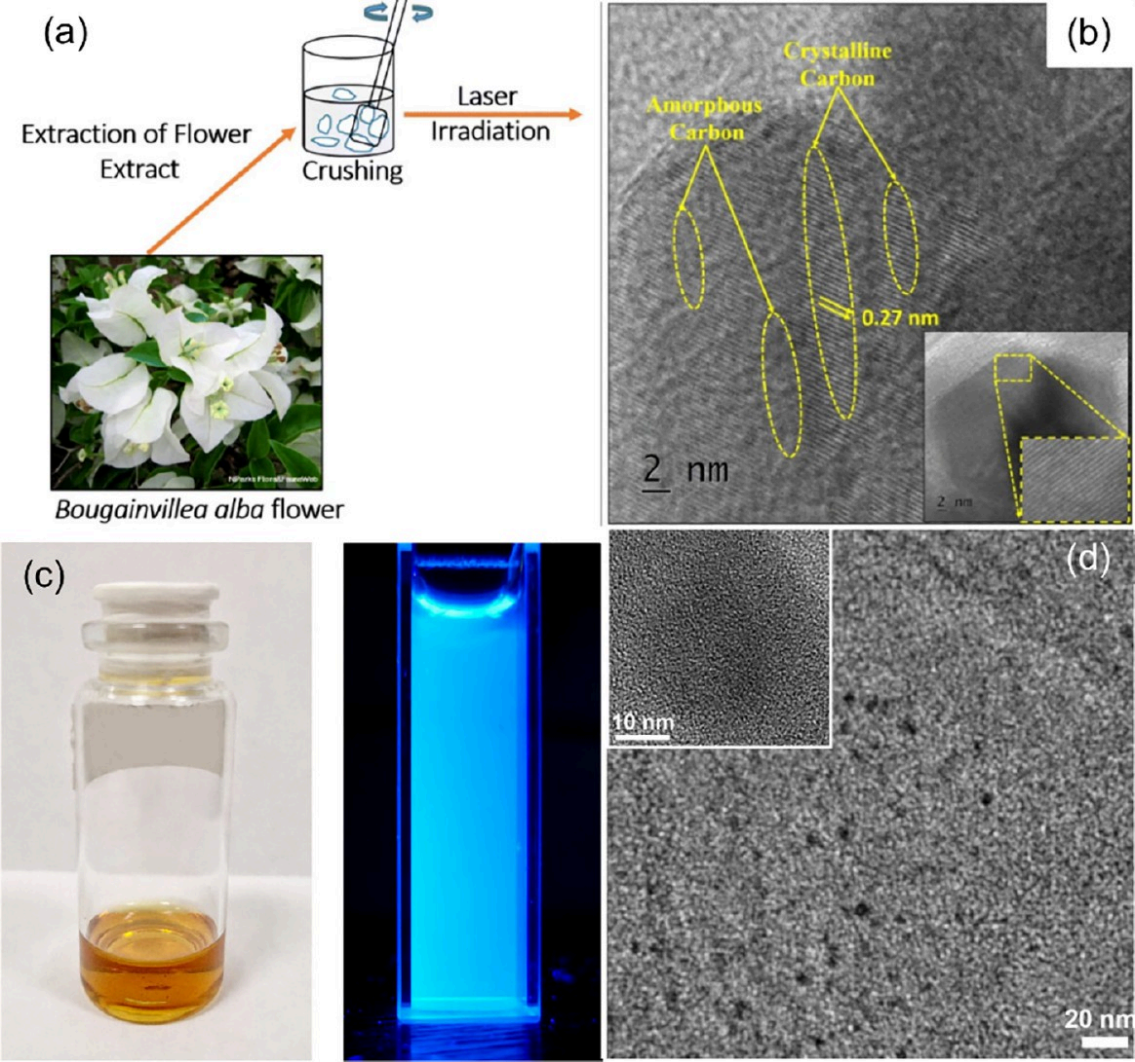
(a) Process of *Bougainvillea alba* flower extract for laser irradiation. (b) HR-TEM of CDs produced from flower extract with amorphous and crystalline features. Inset: Closer look to the crystalline structure of CDs (Reprinted with permission from ref 208. Copyright 2019 Springer Nature). (c) CDs produced from lysine under ambient light (left) and under UV lamp (right) and their (d) corresponding TEM image. Inset: HR-TEM of CDs produced from amino acids (Reprinted with permission from ref 209. Copyright 2024 American Chemical Society).

Apart from carbon allotropes and natural products, ***small molecules*** are also directly employed as carbon sources in the laser-driven synthesis of CDs. To verify this, Astafiev and colleagues^209^ performed a femtosecond laser synthesis of CDs directly from nonaromatic proteinogenic amino acids such as branched-chain amino acids (BCAA), glycine, lysine, glutamine, and arginine, which the authors collectively referred as AA-CDs (amino acid carbon dots). They hypothesized that the femtosecond laser triggered the polymerization and carbonization of amino acids, leading to the formation of CDs, as shown in Figure 17c, for the case of CDs from lysine and their corresponding TEM images (Figure 17d). They also reported that all AA-CDs exhibited similar fluorescence behavior, displaying the excitation-dependent wavelength emission with an intense emission in the blue region. In addition, those CDs prepared from arginine exhibit the highest quantum yield among AA-CDs, equivalent to 7%. They also concluded from their investigation that the choice of amino acid as a carbon precursor dramatically impacts not only their quantum yield but also the rate of carbon dot production. They found that the efficient conversion of amino acids to CDs was higher in lysine, glutamine, and arginine compared with BCAA and glycine. The work of Jin et al.^39^ is another instance where the starting solution is organic molecules for the laser synthesis of CDs. They directly synthesized CDs from heterocyclic aromatic hydrocarbon molecules—benzopyrrole (BTZ), benzothiophene (BT), and benzothiazole (BTA)—dispersed in ethyl acetone using a Nd:YAG laser of 355 nm wavelength. They found that the nitrogen (N) and sulfur (S) atoms doping in the CD’s structure highly depend on the starting molecules. CDs from BTZ possessed N doping due to the existence of N on the aromatic ring of BTZ, CDs from BT had an S doping due to S on the aromatic ring of BT, and CDs from BTA obtained both N and S doping due to the existence of both S and N atoms on the original BTA chemical structure. The researchers referred to the prepared samples based on the doping atom as N-CDs (from BTZ), S-CDs (from BT), and N, S-CDs (from BTA). All the CDs prepared by laser irradiation are highly monodispersed and have a size ranging from 3-4 nm. According to their fluorescence, the doping of both N and S caused a red shift of the maximum emission of CDs from 450 nm (emission from N-CDs) to 475 nm. The researchers also calculated the potential of their dynamic light scattering (DLS) zeta to determine their stability. They found that the colloidal suspension of N, S-CDs is more stable than N-CDs and S-CDs.

Thus, a wide variety of carbon precursors are viable for the synthesis of CDs via PLAL and PLFL. Furthermore, the starting material also plays a crucial role in the cost-effectiveness and sustainability of PLAL and PLFL techniques, and thus, they must be carefully considered. We will address the advantages and disadvantages of different carbon precursors in laser-based synthesis. Biomass waste offers a highly sustainable option, since it is abundant and essentially free with minimal environmental impact. They often contain intrinsic dopants such as N, O, and S which can enhance CD’s optical properties.^210,211^ However, using biomass to make CDs leads to heterogeneity and inconsistency in their physicochemical properties, which pose challenges for reproducibility. Additionally, pre-treatment steps such as drying and carbonization, as well as post-purification processes, are often required, leading to increased energy consumption, cost, and synthesis time. A significant disadvantage of using biomass is the difficulty of upscaling production. This is a major drawback, given that this is one of the primary goals in laser-based synthesis. On the other hand, traditional carbon precursors such as carbon allotropes are better for upscaling production with no or fewer purification processes required. However, they might not be sustainable due to their staple cost and production. Laser-based synthesized CDs from carbon allotropes usually have low fluorescence yield^10^ thus, a post-modification process is often required to enhance their optical properties (further discussed in section 2.3). Laser-synthesized CDs from carbon allotropes can be doped to tune their properties by adding molecular components in the liquids during the laser process or by merely switching solvents.^212^ In addition, small molecules can yield CDs with high fluorescence and excellent doping based on the starting molecular composition.^178,213^ However, the production yield is lower, making them less cost-effective. They can require an extensive purification process. Some molecules have poor laser absorption; thus, one must carefully tune different laser parameters (wavelength or fluence) to process this precursor.

Overall, laser parameters (e.g., fluence, wavelength, irradiation time, pulse width), solvents (organic, inorganic, and ionic), and carbon precursors (biomass, small molecules, and carbon allotropes) are the key factors in optimizing specific properties of CDs for targeted applications. With a combination of these factors such as short-wavelength laser and using organic solvents like ethanol or acetone, laser-synthesized CDs can have higher surface functionality, which is advantageous in attaching different targeting molecules for sensing and targeting bioimaging applications. CDs can achieve enhanced fluorescence, which is highly advantageous for bioimaging (real-time monitoring and tracking) and LEDs by exploring a low laser fluence in amine-containing solvents.

### 2.3 Post-Laser Modifications and Purifications of Laser-Synthesized CDs

Post-laser treatments, such as oxidation,^89^ surface passivation with polymers,^83^ and reduction,^214^ are usually employed to mitigate the limitations imposed by laser-driven synthesis of CDs, such as poor water solubility and low fluorescence yield. Aside from changing the laser parameters, solvent composition, or carbon target, these subsequent treatments can drastically alter the physicochemical properties of the CDs.^89^ Moreover, the post-functionalization process has also been adapted to laser-synthesized CDs to utilize them for their desired applications effectively. The functional groups embedded during laser synthesis make CDs also viable for functionalization processes, which could be either covalent (amidation, esterification, etc.) or non-covalent conjugation (supramolecular interactions such as electrostatic interaction, hydrogen bonding, van der Waals interaction, etc.). The conjugation of different inorganic and organic compounds, biomolecules, and nucleic acids onto the surface of the CDs can tune their properties for enabling specific recognition of cells (quantitative cell labeling), actively targeting of cancer cells (cancer diagnosis and therapy), biosensing, and many more applications.^215^ Although one-step surface passivation and/or functionalization is possible throughout the laser synthesis process, as reported by Hu et al.^82^ and Kang et al.,^216^ the fluorescence yield of these CDs was less than 10% with the optimal synthesis conditions. Purification is necessary to remove unwanted products from the modification processes. Unlike other synthesis methods, laser synthesis typically produces high-purity CDs, requiring purification only when post-laser or intralaser treatments (e.g., dopant compounds were incorporated). At this point of the review, we will enumerate the different post-laser synthesis processes (such as functionalization and their purification methods) of laser-synthesized CDs from various literature.

Because, in some cases, the laser-synthesized CDs exhibit poor stability and low fluorescence, passivation with biocompatible polymers such as PEG has been chiefly utilized, especially in bio applications. Sun et al.^80^ synthesized CQDs through the laser ablation in liquids technique and observed that they did not possess any noticeable fluorescence. After refluxing the CQDs with an aqueous nitric acid solution, they passivated them with diamine-terminated oligomeric PEG (PEG_1500N_) via physical mixing and heating at 120^0^C. After this process, a bright fluorescence was observed from the CQDs, as shown in Figure 18a, with different excitation wavelengths. The authors state that this high fluorescence is due to stabilization of surface energy traps as a result of PEG passivation (Figure 18b). It is important to note that they also used other polymers such as poly­(propionylethyleneimine-co-ethyleneimine) (PPEI-EI). They observed the same significant increase in CD fluorescence. Cortes et al. also observed the same effect when PEG_400N_ was used as the passivating polymer for laser-fragmented CQDs in ethanol. Unlike Sun et al.’s case, CQDs from this work have a slight fluorescence and were enhanced right after PEG passivation, as shown in Figure 18c, and their water solubility. Similarly, Gonçalves et al. observed that the laser-ablated CDs possessed no visible fluorescence. Goncalves and coworkers^83^ activated the carbon nanoparticles through refluxing and PEG_200N_ mixing. The fluorescence of CDs significantly improved after the passivation. They further functionalized the CDs-PEG_200N_ with N-acetyl-l-cysteine (NAC) and observed a blue shift in the emission from 565 nm (CDs-PEG_200N_) to 450 nm. They purified the CDs via solvent extraction with ethyl acetate to remove the free PEG and NAC. They observed that the functionalized CDs-PEG_200N_/NAC were more selective towards mercury ions (Hg^2+^) than other metal ions owing to the addition of NAC, which offers a binding site mainly to Hg^2+^ ions.

**Figure 18:**
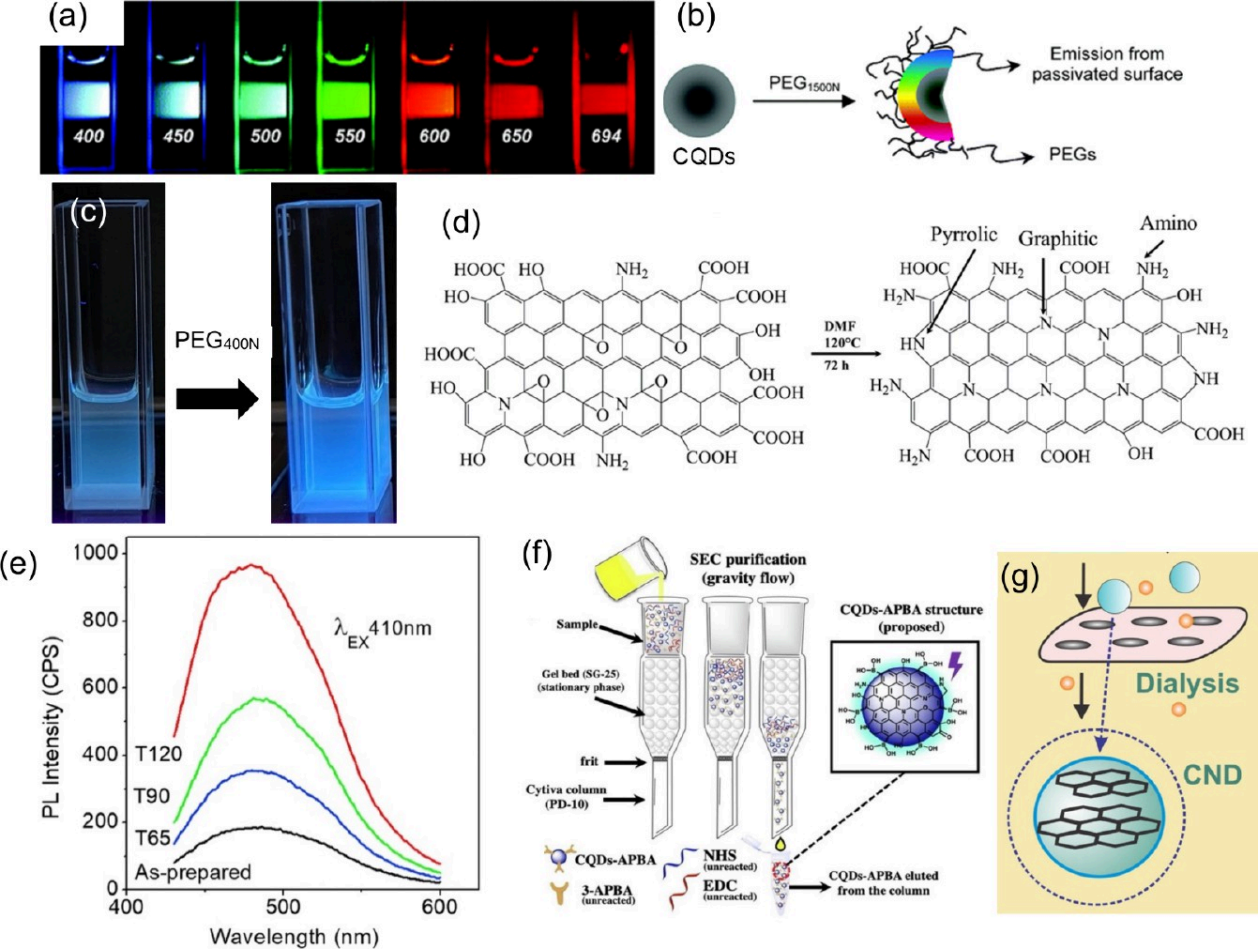
(a) Fluorescence emission of CQDs passivated with PEG_1500N_ at different excitation wavelengths as indicated. (b) Laser-synthesized CDs after passivated with PEG_1500N_ resulting to emission (Reprinted with permission from ref 80. Copyright 2006 American Chemical Society). (c) Fluorescence emission of CQDs under UV lamp before (left) and after passivated with PEG400N (right); (d) GQDs before and after solvothermal treatment in DMF; and (e) fluorescence spectra of GQDs subjected to solvothermal at different temperatures: 65 °C, 90 °C, and 120 °C (Reprinted with permission from ref 214. Copyright 2019 Elsevier Ltd.). (f) SEC purification of CQDs conjugated with boronic acid via amidation reaction (Reprinted with permission from ref 96. Copyright 2024 Elsevier B.V.). (g) Removal of by-products from carbon nanodots (CNDs) solution through dialysis method. (Reprinted with permission from ref 213. Copyright 2022 American Chemical Society).

Laser ablation in liquids with a complementary bottom-up route, such as the solvothermal method as post-laser treatment, has been reported by Novoa-De León et al.,^214^ which intends to increase the fluorescence yield of as-synthesized GQDs from laser ablation in DMF. By using DMF as liquid, the as-synthesized GQD has different oxygen functionalities such as −COOH and −OH on its surface and possessed nitrogen atoms as its dopants (Figure 18d). The as-synthesized GQDs were treated with solvothermal at three different temperatures (65 °C, 90 °C, and 120 °C). Through solvothermal treatment, the as-synthesized GQDs undergo a deoxygenation process, thereby reducing the oxygen content on GQDs while reinforcing the doping of nitrogen atoms (pyrrolic, amino, and pyridinic), as shown in Figure 18d. Owing to this, the fluorescence yield of as-synthesized GQDs initially at 0.6% increases linearly with the solvothermal temperature from 0.91% for 65 °C and 1.74% for 90 °C to 4.05% for 120 °C. Their fluorescence spectra in Figure 18e show that 120 °C treatment exhibits the highest fluorescence for the same emission wavelength of 486 nm. As opposed to the work of Novoa-De León et al., relying on the oxygenated surface of CDs also presents several advantages, as proven by Narasimhan et al.^89^ Using a graphite plate as the target and an aqueous solution of PEG as the solvent, they synthesized GQDs through laser ablation. The as-synthesized GQDs underwent refluxing at 200 °C during two different periods: 20 min and 1 h. They found that the GQDs refluxed at 20 min exhibited a higher QY of 47.16% in comparison to those GQDs refluxed at 1 h with a QY of just 12.8%, which decreases appreciably from the as-synthesized GQDs with a QY of 24.9%. They hypothesized that the change in the QY was due to the introduction of new energy levels from the carboxyls. However, controlling the refluxing time can also significantly affect the QY of GQDs, as observed when refluxing at 1 h. It should also be emphasized that the GQDs refluxed at 1 h showed a higher viability toward the MCF-7 cell line (breast cancer) than as-synthesized GQDs and GQDs refluxed at 20 min. They believed that the high viability was due to more oxygen groups on the surface of the GQDs.

Post-functionalization processes also apply to laser-synthesized CDs. Cortes et al.^96^ modified the carboxylic-rich surface of laser-synthesized CQDs through a simple amidation reaction (EDC/NHS coupling) by attaching amine molecules such as amino phenylboronic acid (APBA) for glucose sensing applications. Boronic acid derivatives, such as APBA are known as glucose receptors. Hence, attaching them to fluorescent compounds such as CQDs can create a fluorescent-based glucose sensor. They utilized size exclusion chromatography (SEC) to remove unreacted entities using Sephadex beads as the stationary phase and water as the eluent (Figure 18f). Concerning purification after the laser synthesis, Kaczmarek et al.^212^ used a simple dialysis method to remove added compounds in the liquid for one-step passivation and functionalization process. They performed laser ablation in a liquid containing branched polyethylenimine (PEI) or EDA to synthesize luminescent CQDs. Using a dialysis kit with a molecular weight cutoff (MWCO) of 500 Da (Dalton), they dialyzed CQDs in PEI and EDA against deionized water for five consecutive days. Due to the high-energy pulsed laser directed to the solution, the laser irradiation can affect free PEI and EDA molecules, changing their chemical structure and creating new side by-products such as molecular fluorophores. They state that the molecular fluorophores could mask the actual signal of the CQDs passivated with PEI and CQDs passivated with EDA, leading to a significant error in the QY calculation. Astafiev et al.^213^ also employed the dialysis method against water to purify CDs synthesized by femtosecond laser irradiation of aromatic compounds such as benzene and pyridine. Using 2 kDa MWCO dialysis, they retained the formed CDs and removed the side products produced by laser irradiation (Figure 18g). This shows that purification becomes more relevant when molecular compounds are added directly during the laser synthesis of CDs.

## 3 Applications of Laser-Based Synthesized CDs

The target and liquid versatility of PLAL and PLFL syntheses to fabricate CDs with a wide variety of superior and excellent physicochemical properties can be futile without exploring their capability in relevant application areas. Thus, in this section, we will delve into various published literature focusing on the applicability of laser-synthesized CDs as fluorescence bioimaging agents, optical-based sensors, and antibacterial and anticancer. Furthermore, this review discusses the applicability of these CDs to other bio-related applications and beyond.

### Laser-Synthesized CDs for Fluorescence Bioimaging

Due to their strong intrinsic fluorescence emission and excellent biocompatibility, CDs have been continuously applied as fluorescent probes in cellular imaging for cancer diagnosis and *in vivo* imaging. Cui et al.^187^ synthesized homogenous CQDs through dual-beam laser ablation of a low-cost carbon cloth in DMSO. They tested the inherent cytotoxicity of the prepared CQDs on HeLa (human cervical cancer) cells via 3-(4,5-dimethyl-2-thiazolyl)-2,5-diphenyl-2-H-tetrazolium bromide (MTT) viability assay. They found that the CQDs have very low cytotoxicity for HeLa cells even at the concentration of 1000 *μ*g/mL CQDs (Figure 19a). To visualize the uptake of CQDs by HeLa cells, they used confocal microscopy imaging operated at an excitation wavelength of 405 nm. They found that the CQDs were well-internalized by the cells and showed good biocompatibility (Figure 19b). Astafiev et al.^209^ employed a femtosecond laser to synthesize CDs from amino acids as a fluorescent agent in A549 (lung adenocarcinoma) cells. After treating A549 cells with CDs from lysine (Lys-CDs), they measured the percentage of dead cells via acridine orange (AO) and propidium iodide (PI) staining. The result showed that Lys-CDs do not cause cell death and thus are highly biocompatible. Confocal fluorescent images of A549 cells showed the effectiveness of Lys-CDs as a fluorescence bioimaging agent (Figure 19c). Doñate-Buendía et al.^10^ also investigated the fluorescence cellular imaging capability of laser-synthesized CQDs from glassy carbon powder in PEG_200_ via a PLFL flow-jet configuration system. They found that the cellular internationalization of CQDs in the OEC (oral epithelial cells), which took approximately 1 min, was faster compared to the HT-29 (colon adenocarcinoma) cell line, which took about 10 min (Figure 19d–19e). They further investigated the photostability of CQDs, where they tracked the intensity of CQDs in comparison to commercial fluorescent markers, such as Alexa Fluor 488. The photobleaching of Alexa Fluor 488 was observable only at 7 min, while it was observable for 2 h for CQDs. In the case of *in vivo* imaging, Narasimhan and co-workers^89^ applied ns-PLAL synthesized GQDs from highly oriented pyrolytic graphite (HOPG) to an euthanized mouse. They mixed the prepared GQDs with acrylamide gel and implanted them subcutaneously in the thoracic region of the mouse. Using an in-house optical imaging system, they acquired bright red fluorescence from the implanted GQDs excited at 530 nm using a 590 nm emission filter as shown (Figure 19f). Furthermore, Jumardin et al.^207^ synthesized CDs by laser fragmentation of tea in toluene and used them for bioimaging of a zebrafish. With a dose of 0.001 cc/mL, they injected CDs into four zebrafish at four different points (tail, intestinal, dorsal, and gill) (Figure 19g). They found that the CDs accumulated in the eye cavity of the zebrafish (for the case of intestinal, dorsal, and gill injection points) emitted blue fluorescence, as observed by a confocal laser microscope. They discovered an observable fluorescence for gill, intestinal, and dorsal injection (Figure 19h-i, 19h-ii, 19h-iii) but none for tail injection points (Figure 19h-iv).

**Figure 19:**
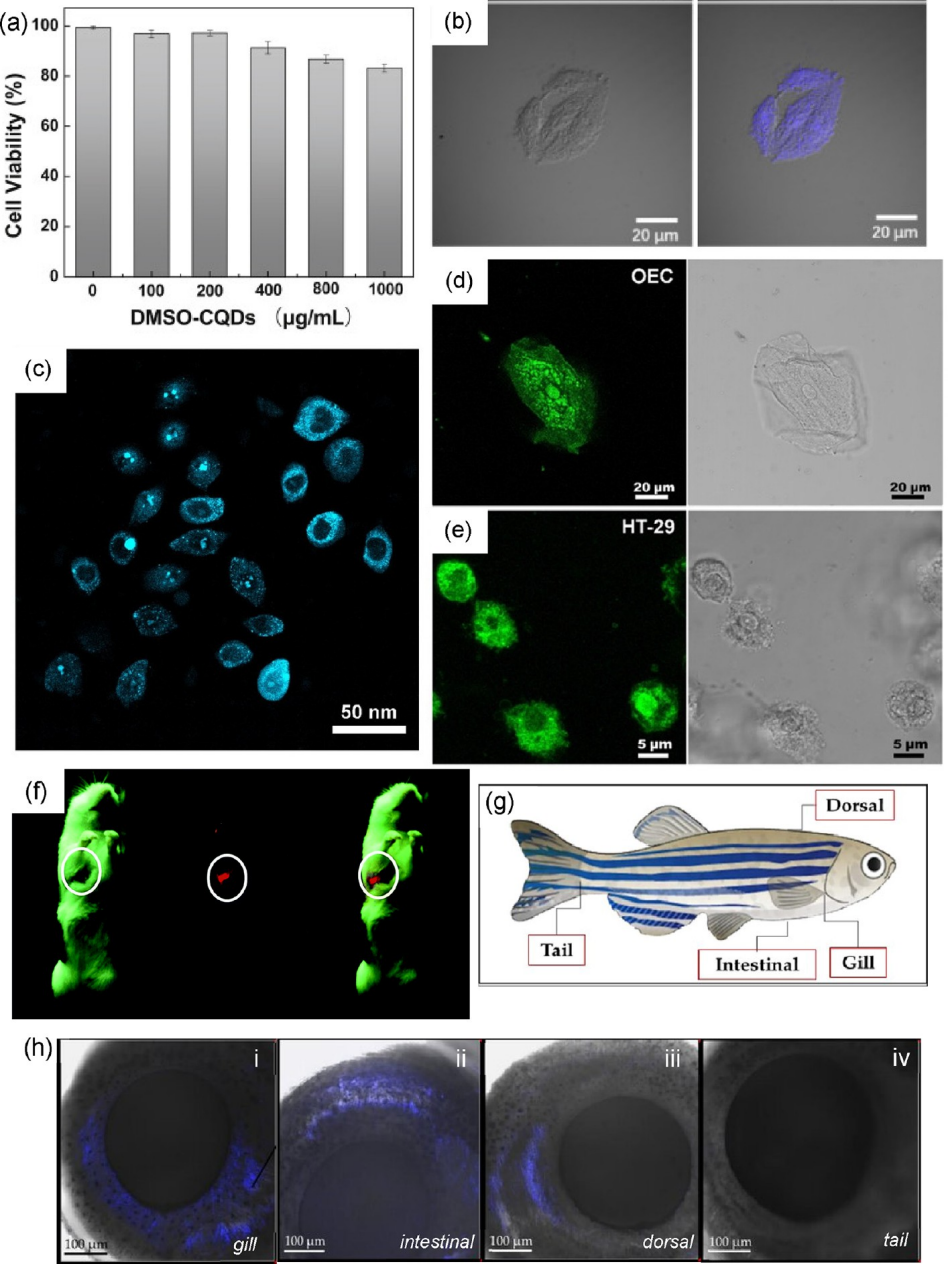
(a) Cell viability test of DMSO-CQDs at different concentration in *μ*g/mL; (b) Bright-field and the corresponding overlay images of HeLa cells incubated with CQDs from confocal microscope (Reprinted with permission from ref 187.Copyright 2020 Elsevier Ltd.); (c) Confocal microscope image of A549 cells after addition of Lys-CDs (Reprinted with permission from ref 209. Copyright 2024 American Chemical Society); Confocal fluorescence (left) and bright-field images (right) of (d) OEC and (e) HT-29 cells incubated with CQDs (Reprinted with permission from ref 10. Copyright 2018 American Chemical Society); (f) *In vivo* imaging of a euthanized mouse implanted with GQDs-acrylamide gel (red) in the thoracic region as indicated by the white circle (Reprinted with permission from ref 89.Copyright 2017 Royal Society of Chemistry); (g) Different region of CDs injection to a zebrafish; (h) Blue luminescence of CDs located in the eye of a zebrafish after (i) gill, (ii) intestinal, (iii) dorsal, and (iv) tail injection. (Reprinted with permission from ref 207.Copyright 2023 Jurnal Ilmu Fisika).

### Laser-Synthesized CDs as Optical-Based Sensors

CDs’ intrinsic fluorescence and sensitivity also made them an alternative to fluorescent-based sensors for detecting pH, temperature, various metal ions, and different analytes. Kang et al.^216^ synthesized amino functionalized GQDs (FGQDs) from graphite flakes in high-purity ethanol with polypyrrole by a one-step pulsed fragmentation process using an Nd:YAG laser system. They utilized the prepared FGQDs as Fe^3+^ metal ion fluorescent-based sensors. They found a decrease in the fluorescence at 450 nm as the concentration of Fe^3+^ metal ions increased, as shown in their fluorescence spectra (Figure 20a). The prepared FGQDs have good sensitivity toward Fe^3+^ ions with a limit of detection (LOD) measured to be 5 *μ*M. FGQDs also provide good selectivity to Fe^3+^ ions where the fluorescence quenching is stronger than any other metal ions (Figure 20b). Goncalves et al.^83^ also studied the selectivity and sensitivity of laser-synthesized CDs to detect Hg^2+^ ions. They prepared CDs via direct laser ablation of a carbon target immersed in water using a 248 nm UV pulsed laser. After functionalization with PEG200 and N-acetyl-l-cysteine, they discovered that the CDs enhanced their emission and are now sensitive to Hg^2+^ ions; that is, the fluorescence decreases upon adding Hg^2+^ ions at a micromolar concentration. They studied the selectivity of the prepared functionalized CDs to other ions such as Cd^2+^, Ni^2+^, Zn^2+^, Ca^2+^, and Co^2+^ ions. They found that these ions did not significantly influence the fluorescence, confirming the selectivity of the CDs.

**Figure 20:**
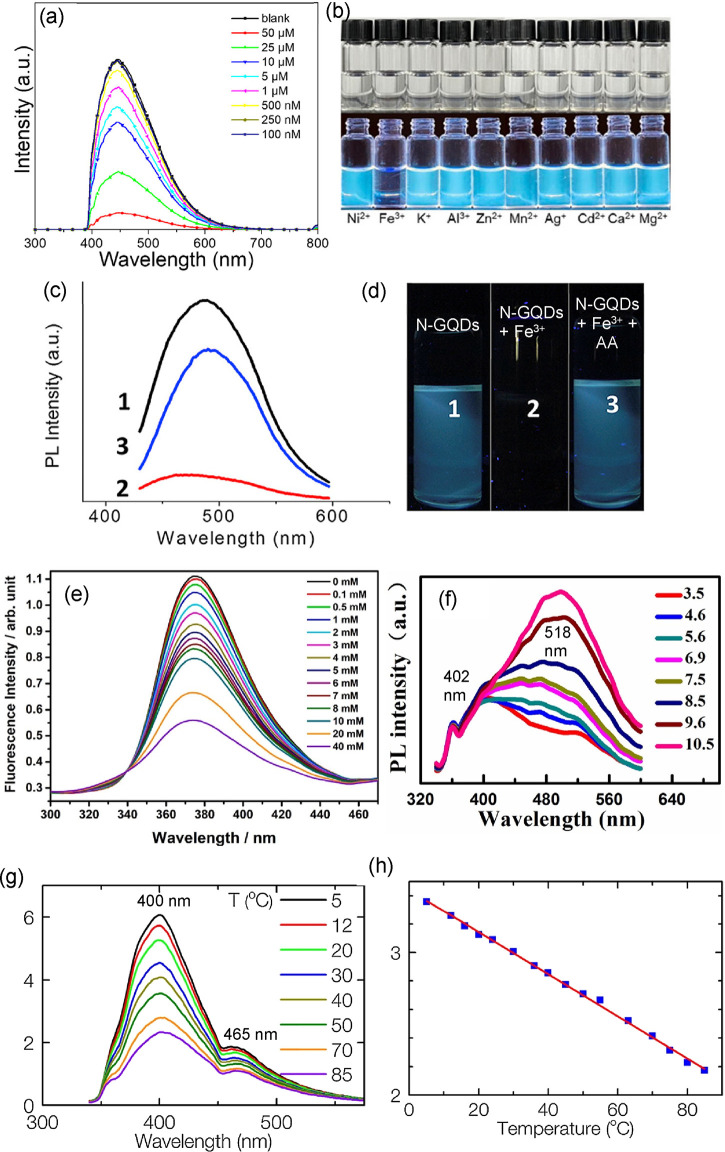
(a) Fluorescence spectra of FGQDs at increasing Fe^3+^ concentration centered at 450 nm; (b) FGQDs fluorescence emission under UV lamp with the addition of different metal ion species as indicated (Reprinted with permission from ref 216.Copyright 2022 American Chemical Society); (c) Fluorescence spectra of (1) N-GQDs, (2) N-GQDs after addition of Fe^3+^ ions, and the (3) fluorescence recovery after addition of ascorbic acid and (d) their corresponding fluorescence emission under UV lamp (Reprinted with permission from ref 214.Copyright 2019 Elsevier Ltd.); (e) Fluorescence spectra of CQDs-APBA after addition of glucose at increasing concentration (Reprinted with permission from ref 96.Copyright 2024 Elsevier B.V.); (f) Fluorescence spectra of N-doped CDs with two maximum emissions (402 and 518 nm) at different pH (Reprinted with permission from ref 217.Copyright 2017 Elsevier B.V.); (g) Fluorescence spectra of laser-synthesized CDs with two maximum emissions (400 and 465 nm) at increasing temperature (°C) and (h) their corresponding linear relationship curve (Reprinted with permission from ref 218.Copyright 2017 Elsevier B.V.).

The sensing capability of laser-prepared CDs is limited not only to metal ions but also to different molecules and analytes. Novoa-De León et al.^214^ developed a fluorescence on–off-on detection probe for Fe^3+^ ions and ascorbic acid (AA) based on laser-synthesized N-doped GQDs (N-GQDs) fabricated via PLAL using a 532 nm Nd:YAG laser system. They found that the fluorescence of N-GQDs was sensitive to Fe^3+^ ions, decreasing its fluorescence intensity (fluorescence off). Furthermore, they discovered that the fluorescence of the N-GQDs/Fe^3+^ system can be slowly recovered (fluorescence on) after reaction to AA (Figure 20c,d) with a good sensitivity up to 30.96 nM (LOD). The selectivity of the N-GQDs/Fe^3+^ system showed that the fluorescence recovery is much stronger with AA than with other biomolecules (l-cysteine, uric acid, glucose, and dopamine). Moreover, Cortes et. al.^96^ also demonstrated the glucose-sensing capability of laser-synthesized CQDs. They synthesized CQDs via ns-PLFL of carbon black powder in ethanol. The CQDs were functionalized with amino phenylboronic acid (APBA) as a glucose receptor. They found that the APBA-functionalized CQDs (CQDs-APBA) exhibit a decrease in fluorescence after adding glucose with a sensitivity of 165 *μ*M (LOD) as shown in Figure 20e. They tested the selectivity of CQDs-APBA by incorporating different saccharides (fructose, sucrose, maltose, and lactose), ions (K^+^, Cl^–^, and Na^+^), and other interfering biomolecules (glutathione, ascorbic acid, urea, cysteine, and phenylalanine). They found that the CQDs-APBA have a good selectivity glucose. They further tested the sensing capability of the CQDs-APBA with real analytes such as human saliva and found a good reproducibility of the method.

Aside from metal ions and biomolecules, laser-synthesized CDs have also been applied as fluorescent-based pH and temperature sensors. For instance, Xu and colleagues^217^ produced N-doped CDs via one-step fs-PLFL of graphite powder in aminotoluene for ratiometric pH sensing. They implemented a ratiometric sensing method by tracking the intensity ratio of two emission peaks from the CDs: 402 and 518 nm. The emission peak at 518 nm increases significantly compared to the 402 nm emission when increasing the pH (Figure 20f). They constructed two linear standard curves for different pH values: (1) from pH = 3.5–7.5 and (2) from pH = 7.5–10.5. Moreover, Nguyen et al.^218^ proposed laser-synthesized CDs as ratiometric temperature sensors based on the intensity ratio of two emission peaks from CDs when excited at 320 nm: 400 and 465 nm. They found that CDs exhibited a decrease in fluorescence for both emission peaks as the temperature increased (Figure 20g). They found a good linear relationship between the intensity ratio (*I*_400_/*I*_465_) and the temperature in a range from 5 to 85 °C with a linear correlation of 0.998 (Figure 20h). The CDs showed a sensitivity to temperature of 1.48% change per °C over the range 5–85 °C. Thus, they demonstrated that laser-synthesized CDs can be a promising alternative as an excellent ratiometric nanothermometer.

### Laser-Synthesized CDs as Antibacterial and Anticancer Agents

CDs and other NPs displayed promising antibacterial properties to address the growing bacteria resistance to conventional antibiotics. CDs’ surface reactivity and functionality enable them to diverge from the antibacterial mechanism of established antibiotics.^219^ The report from Jonathan et al.^220^ showed that laser-synthesized CDs, like many other synthesis approaches, can be a good alternative as an antibacterial agent. The prepared CDs were fabricated via the PLAL method, employing an Nd:YAG laser system (1064 nm) with high-purity graphite plates as targets. They studied the antibacterial effect of CDs ablated at different solvents: deionized water (DIW), chitosan solution (CS), and an EDA solution. They evaluated the antibacterial response of the CDs against Escherichia coli (E. coli) bacteria via the Kirby Bauer disk diffusion test. This test was employed to assess the resistance or sensitivity of bacteria to CDs, indicated by the observed diameter of the inhibition zone (DIZ) in mm. The larger the DIZ, the higher the sensitivity of a particular substance. Using terramycin and DMSO solution as their positive and negative control, respectively, they found that CDs prepared in EDA (Figure 21a) produced the largest DIZ (11.15 mm) compared with those prepared in DIW (7.5 mm) and CS (7.6 mm) (Figure 21b). According to them, the difference in DIZ among CDs is due to the smaller size of CDs in EDA compared to the other CDs, which in turn increases their reactivity, increasing their antibacterial effect. The work by AlMalki et al.^95^ is another case that proves the antibacterial activity of laser-synthesized CDs. Their study fabricated CDs by ablating a graphite target in deionized water at different laser energies (60, 80, 160, and 220 mJ). They evaluated their antibacterial effects against E. coli and Staphylococcus aureus (S. aureus) bacteria. The antibacterial activity of those CDs was tested using an agar well diffusion assay and evaluated by calculating the DIZ. Using deionized water as the negative control (A), they found that CDs prepared at 220 mJ (E) had a larger DIZ to both bacteria compared to CDs at 60 mJ (B), 80 mJ (C), and 160 mJ (D) (Figure 21c-d). They measured the DIZ of CDs at 220 mJ to be about 34 ± 1.0 mm for S. aureus and about 31 ± 1.5 mm for E. coli bacteria. The discrepancy of DIZ among CDs is due to the CDs’ concentration rather than the CDs’ size. According to them, a laser energy of 220 mJ produced the highest concentration of CDs compared to those relatively lower laser energies.

**Figure 21:**
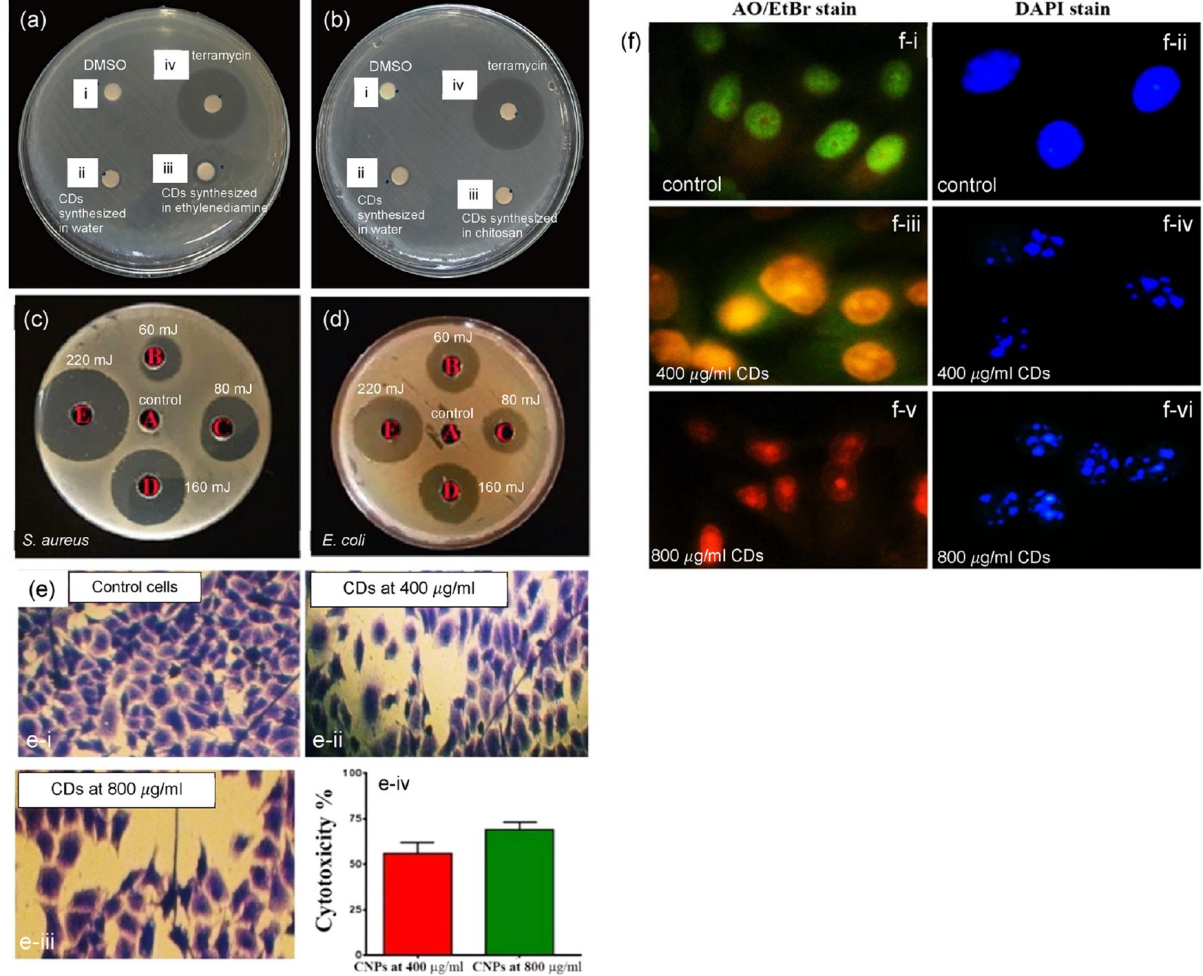
(a) Kirby Bauer diffusion test results of (iii) laser-synthesized CDs in EDA solution in comparison to (i) DMSO only as negative control, (ii) CDs synthesized in deionized water, and (iv) terramycin only as positive control; (b) Kirby Bauer diffusion test results of (iii) laser-synthesized CDs in chitosan solution in comparison to (i) DMSO only as negative control, (ii) CDs synthesized in deionized water, and (iv) terramycin only as positive control (Reprinted with permission from ref 220.Copyright 2023 Elsevier B.V.); Agar well diffusion assay results of CDs synthesized at different fluences: (B) 60 mJ, (C) 80 mJ, (D) 160 mJ, and (E) 220 mJ with (A) deionized water as the control, against (c) S. aureus and (d) E. coli bacteria (Reprinted with permission from ref 95.Copyright 2022 John Wiley & Sons, Inc.); (e) Microscopy images of MCF-7 cells: (i) untreated and treated with CDs at different concentrations: (ii) 400 *μ*g/mL and (iii) 800 *μ*g/mL and their (iv) corresponding viability assay result; (f) MCF-7 cells (i-ii) untreated and treated with CDs of two concentrations: (iii-iv) 400 *μ*g/mL and (v-vi) 800 *μ*g/mL, stained with AO/EtBr (left) and DAPI (right). Scale bar 10 *μ*m (Reprinted with permission from ref 221.Copyright 2020 Institute of Physics Publishing Vietnam Academy of Science & Technology)

Furthermore, the laser-synthesized CDs have also been tested as an anticancer agent with superior biocompatibility in both in vitro and in vivo. The work by Khashan et al.^221^ showed the anticancer activity and high *in vivo* biocompatibility properties of CDs prepared by PLAL. They employed an Nd:YAG laser system (1064 nm) to irradiate graphite pellets in deionized water, producing CDs via the PLAL approach. To evaluate their anticancer response, they treated the MCF-7 (breast cancer) cell line with CDs of two different concentrations: 400 and 800 *μ*g mL^–1^. Under an inverted phase microscope, they found a significant alteration of MCF-7 cell morphology and a reduction in cancerous cells compared to the control (Figure 21e-i, e-ii, and e-iii). Using an MTT assay to assess their cytotoxicity, they found that CDs at both concentrations are cytotoxic and inhibit MCF-7 cell proliferation after 72 h of treatment. Notably, the 800 *μ*g mL^–1^ of CDs exhibits less anti-proliferative effect on the mentioned cell line than 400 *μ*g mL^–1^ of CDs (Figure 21e-iv). To know the CDs mechanism of action for apoptosis (cell death), they further stained the MCF-7 cell line with dual acridine orange/ethidium bromide (AO/EtBr) for morphological alterations of the cell nucleus and 4′,6-diamidino-2-phenylindole (DAPI) for cell nucleus observation. Under a fluorescence microscope, the non-apoptotic cells emit green fluorescence (control, f-i). In contrast, the apoptotic cells emit orange or red fluorescence (400 *μ*g mL^–1^ of CDs, f-iii, and 800 *μ*g mL^–1^ of CDs, f-v) (Figure 21f). Interestingly, they observed that the nuclei of treated cells stained with DAPI (f-iv and f-vi) showed chromatin condensation as opposed to normal nucleus morphology (f-ii), as shown in Figure 21f. They attributed this observation to the induced reactive oxygen species (ROS) by CDs which resulted in DNA damage. Moreover, they also assessed the toxicity of the prepared CDs in mice (*in vivo*). After 4 weeks, they found that administering CDs (400 and 800 *μ*g mL^–1^) to animals did not induce histopathological modifications, specifically in the liver, kidney, spleen, and lung. Therefore, laser-synthesized CDs are also viable both in vitro and in vivo, making them a valuable asset in nanomedicine.

Moreover, Habiba et. al.^88^ designed a hybrid structure composed of laser-synthesized GQDs and silver NPs as an effective chemotherapeutic drug delivery system for doxorubicin (DOX) and a photosensitizer in chemo-photodynamic therapy (combination of chemotherapy and photodynamic therapy). They synthesized the hybrid structure of Ag NPs and GQDs from the laser fragmentation of a mixture of Ag powder and PEG bis­(3-aminopropyl) terminated in benzene. The PEGylated Ag-GQDs were then loaded with DOX through *π*-*π* stacking and hydrophobic interactions. The cytotoxicity of Ag-GQDs/DOX nanoconjugate was evaluated using human cervical cancer cells (HeLa) and prostate cancer cells (DU145) via the 3-(4,5-dimethylthiazol-2-yl)-5-(3-carboxymethoxyphenyl)-2-(4-sulfophenyl)-2H-tetrazolium (MTS) assay. They found that the nanoconjugates are more toxic to the mentioned cells compared to GQDs alone, DOX alone, and Ag-GQDs without DOX. To test their efficacy in photodynamic therapy, they irradiated Ag-GQDs/DOX with a 425 nm LED lamp. They found a 75% reduction in cancer cell viability using the combination of both therapeutic approaches compared with treatment with Ag-GQDs/DOX alone, which has a 68% reduction. Thus, laser-synthesized GQDs can also be utilized as a multifunctional therapeutic modality for cancer therapy.

### Other Reported Applications of Laser-Synthesized CDs

The previously mentioned applications of CDs produced via laser synthesis as sensors, bioimaging agents, and antibacterial and anticancer agents were only the tip of the iceberg. The broad reach of laser-synthesized CDs is proven to be not limited to the mentioned applications, but they have also been implemented in electrocatalysis,^202^ printing applications,^222^ lubricant additives,^39^ tissue scaffolding,^174^ and Alzheimer’s disease alleviation.^94^ Calabro et al.^202^ synthesized GQDs via ns-PLAL from carbon nano onion as the target, immersed in different solvents (water, ammonia, ethylenediamine, and pyridine). The GQDs synthesized by laser in water, ammonia, ethylenediamine, and pyridine were called Ox-GQDs, N_A_-GQDs, N_E_-GQDs, and N_P_-GQDs, respectively (as mentioned in section 2.2.2). For the electrochemical study, they employed Ag/AgCl as the reference electrode, a platinum (Pt) coil as the counter electrode, and a GQDs/carbon black composite as the working electrode. They evaluated the electrocatalytic performance of these GQDs for the efficient generation of hydrogen peroxide (H_2_O_2_) through oxygen (O_2_) reduction compared to the conventional catalyst, Pt/C (platinum on carbon) as reference and carbon black only (without GQDs) as the control. They reported that the H_2_O_2_ production of all three N-doped GQDs (N_A_-GQDs, N_E_-GQDs, and N_P_-GQDs) is near 80%, whereas that of Ox-GQDs is only at 60%. Thus, laser-synthesized GQDs can also be used as efficient catalysts for H_2_O_2_ production. Furthermore, Bagga et al.^222^ demonstrated the application of laser-synthesized CDs from a graphite target in water to printing applications. They employed the ligand-free and high purity of the laser-synthesized CDs as a relevant factor in fabricating nanofluid ink for inkjet printing applications. The viscosity of the ink must be within the range of 1–20 mPa·s to eliminate any undesirable flow (excess fluid or printer blockage). By mixing ligand-free CDs with an aqueous solution of glycerol and isopropyl alcohol, they produced a homogeneous and stable suspension of nanoinks with the required viscosity for printing between 0.89 and 12 mPa·s. In addition, laser-synthesized CDs were applied as lubricant additives, specifically to Polyalphaolefin 10 (PAO 10), as demonstrated by Jin and colleagues.^39^ PAO is commonly used as a lubricant in the industrial and automotive industries. Jin et al. synthesized three kinds of CDs with different doping, namely, N-doped CDs, S-doped CDs, and N,S-doped CDs via laser fragmentation of aromatic hydrocarbon molecules. Then, they evaluated the tribological performance of these CDs by measuring friction coefficient (COF) and wear volume after adding them (1 wt %) to PAO base oil. Note that wear volume is defined as a material loss on a surface due to friction.^223^ Surprisingly, they found that N and S-doped CDs + PAO have a lower COF (0.093) compared to PAO only (0.650), N-doped CDs + PAO (0.138), and S-doped CDs + PAO (0.106). Additionally, N, S-doped CDs + PAO composites dramatically reduce the wear volume by up to 92%. These results perfectly complemented the scanning electron microscopy (SEM) images of four worn surfaces lubricated by PAO only (Figure 22a-i), N-doped CDs + PAO (Figure 22a-ii), S-doped CDs + PAO (Figure 22a-iii), and N, S-doped CDs + PAO (Figure 22a-iv), respectively. Remarkably, the abrasive and adhesive wear substantially diminishes from the surface with PAO compared to the surface with N,S-doped CDs + PAO. Therefore, the addition of CDs to PAO increases its lubrication efficiency, as observed by the reduction of the COF and wear volume. Another case where laser-synthesized CDs were used as additives can be found in the work by Cutroneo et al.^174^ Instead of lubricant, they added PLAL-synthesized CDs from a vegetable carbon target immersed in PBS to polycaprolactone (PCL). PCL is a widely used polymer in tissue engineering owing to its biocompatibility and biodegradability. PCL-containing fluorescent CDs (Figure 22b-ii and b-iii) can be helpful and ideal for the non-invasive monitoring of scaffolds. Moreover, they hypothesized that the PCL-CD composites can also assist fluorescent cell staining on scaffolds.

**Figure 22:**
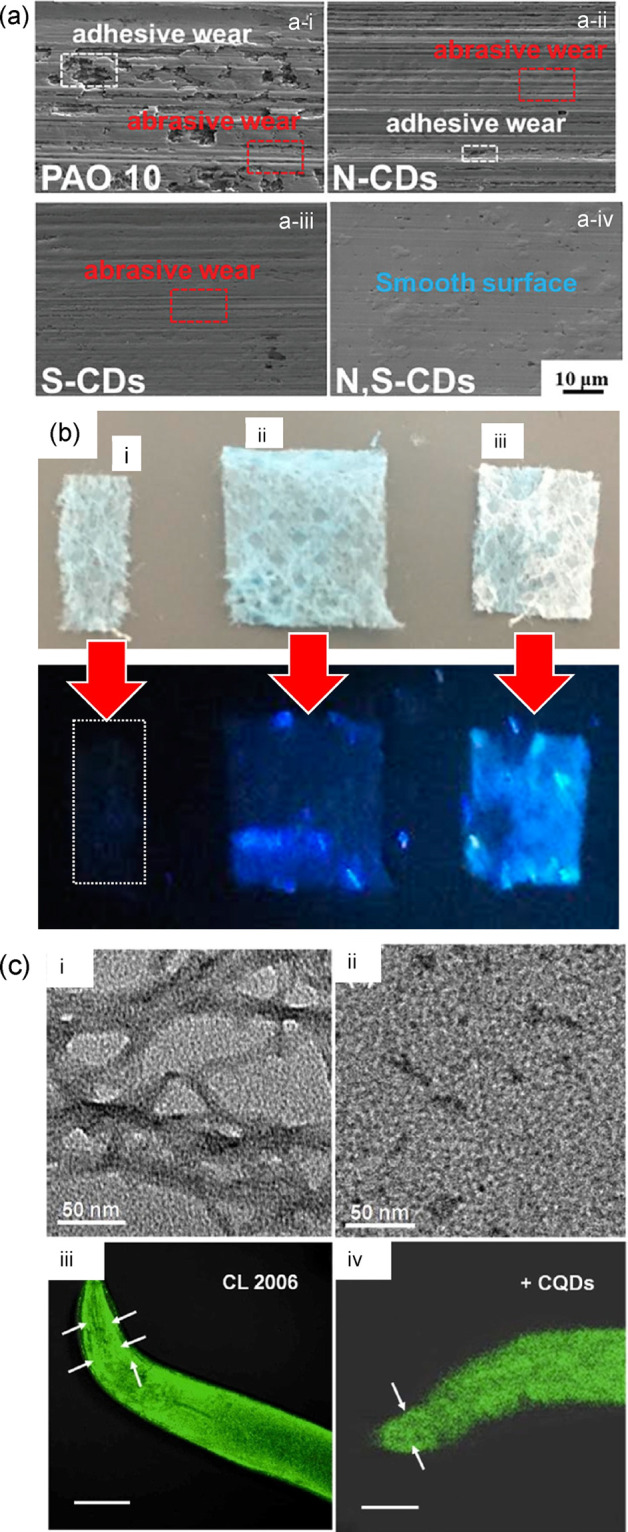
(a) SEM images of worn surfaces lubricated by (i) PAO only, (ii) N-doped CDs + PAO, (iii) S-doped CDs + PAO, and (iv) N,S-doped CDs + PAO (Reprinted with permission from ref 39.Copyright 2024 John Wiley & Sons, Inc.); (b) PCL (i) without and wet with CDs for (ii) 30 mins and (iii) 60 mins (Reprinted with permission from ref 174.Copyright 2024 Multidisciplinary Digital Publishing Institute); (c) TEM images of *β*-amyloid peptides (A*β*42) (i) alone and incubated with (ii) 40 *μ*g/mL of CQDs and ThS fluorescence images of Caenorhabditis elegans nematodes (iii) untreated and (iv) treated with CQDs. Note: White arrows indicate A*β* plaques in nematodes. Scale bars are 20 *μ*m. (Reprinted with permission from ref 94.Copyright 2022 Elsevier B.V.).

Finally, the laser-synthesized CDs are known to inhibit the amyloid-*β* (A*β*) peptides aggregation, which is one of the effective techniques to ease the symptoms of Alzheimer’s disease (AD), as proven by the work of Li et al.^94^ Using *β*-amyloid peptides (A*β*42), they observed the inhibition effect of CQDs through the TEM of A*β*42 at different concentrations. The A*β*42 incubated alone formed a reticular fibril structure as shown in Figure 22c-i but became shorter and shorter after incubating with CQDs at various concentrations: 10 *μ*g/mL, 20 *μ*g/mL, and 40 *μ*g/mL (Figure 22c-ii), which further indicates the inhibitory capability of CQDs. Furthermore, they extended their study about the inhibitory effect of CQDs toward A*β* peptides in *in vivo* using Caenorhabditis elegans CL2006 nematodes as the model for AD study. Utilizing the thioflavin S (ThS) staining assay to determine amyloid plaques of nematodes, the untreated CL2006 clearly showed an apparent aggregation of A*β* peptides (white arrows) based on the higher fluorescence as shown by its confocal microscopy image (Figure 22c-iii). After feeding CL2006 with 10 *μ*g/mL, the aggregation of A*β* was significantly reduced, as indicated by a low fluorescence intensity (Figure 22c-iv).

In summary, laser-synthesized CDs can be a promising alternative for various applications, exhibiting performance that is comparable to—or even superior—to those produced by conventional chemical methods. As mentioned above, they have demonstrated excellent sensitivity and selectivity in bio- and chemical sensing. They exhibited good capability as fluorescent agents in bioimaging with good photostability. They have also shown notable anticancer and antibacterial properties. They have been explored in other innovative areas such as additive manufacturing. Other than the applications discussed in this review, applying laser-synthesized CDs in other fields could further unlock their full potential. This can be realized with the aid of interdisciplinary collaborations for breakthroughs and applications. For example, tailoring the surface functionality of laser-synthesized CDs to be an effective drug delivery system (DDS) in cancer therapy demands expertise from both chemistry and surface engineering. Moreover, understanding the mechanism of cellular uptake, targeting, and toxicity of these DDS (where laser-synthesized CDs serve as the primary component—needs collaboration with experts in biotechnology and nanomedicine. Integrating data science and computational modeling of laser-synthesized CDs can also provide valuable theoretical insights into the design and tuning of their physicochemical properties. With the growing popularity of laser-based synthesis methods, the application of laser-synthesized CDs can soon be expanded through collaboration and combination among various fields, leading to new nanotechnology developments.

## 4 Conclusions and Perspectives

Over these past few years, CQDs and GQDs have made their mark in various sectors ranging from bio-related applications, catalysis, and optoelectronics to harvesting solar energy. There have been several synthesis routes for these NPs; however, some require complicated experimental systems, harsh experimental conditions, toxic chemical by-products, laborious synthesis processes, and energy inefficient. Hence, it is desirable to develop a synthesis method that is easy to implement, has less manual operation, an environmentally friendly and user-friendly production process, and has cost-effective experimental setups. In this article, we showed that the laser-based synthesis in liquids such as PLAL and PLFL offers several advantages, such as their capability to create high-purity CQDs and GQDs, which eliminates time-consuming purification processes, their simplicity and straightforwardness, their versatility to various experimental configurations, their green synthesis approach, and last, their possibility to do upscale production which is viable toward industrial and economic requirement. Both PLAL and PLFL can control and alter the chemical, optical, electronic, electrical, magnetic, and morphological properties of CQDs and GQDs by tuning different variables such as the laser parameters (laser fluence, repetition rate, laser wavelength, irradiation time, pulse width), liquid environment (inorganic, organic, ionic, viscous liquids), and carbon precursors (carbon allotropes, biomass, small molecules). The option to use biomass and small molecules as starting precursors shows that the laser-synthesis in liquids approach is not limited only to the classical “top-down” approach but also the “bottom-up” approach. Apart from these primary variables, there is an abundance of secondary factors to play with to achieve the desired CQDs and GQDs properties through PLAL and PLFL. For instance, changing the temperature of the liquid, mixing different types of solvents, addition of optical elements, pre-treatment of carbon precursors, and incorporation of active molecules during laser treatment could extend the library of laser-synthesized CQDs and GQDs possessing various properties. All this could broaden our knowledge and understanding of the mutual relationship between those primary and secondary variables, the geometry, composition, and structure of CQDs and GQDs, and their correlation and effects on their physicochemical properties. This could also resolve the current challenge that CQDs and GQDs synthesized from this technique face, such as their low fluorescence yield, especially in red or near-infrared emission. To uphold their role as alternatives to semiconductor QDs, they must possess high red- and/or near-infrared fluorescence. This challenge remains a hindrance not only to the laser synthesis technique but also to other synthesis routes. With the combined effort of different synthesis routes, both theoretically and experimentally, red and near-infrared CQDs and GQDs fluorescence will not be as rare as they are right now in the near future.

Another challenge that this method faces is the production upscaling required to compete with industrial NPs production processes. Today’s production rate of NPs has already met the bio-applications standard but further research must be done with regards to their industrial-scale manufacturing and commercial translation.^53^ In recent years, several companies specializing in NPs production via LSPC have already started to emerge. One of those companies generates NPs of high purity and stability without the use of any surfactants and additives and markets them as bio-active.^224^ Another company aims to implement laser-based synthesis NPs in industry with technological and economic feasibility; thus, they developed light hydrodynamic pulse (LHDP) technology. This technique makes use of the light-acoustic shockwave instead of the traditional plasma generated in conventional PLAL. They generated a 200-fold increase in the production and cost reduction.^225^ Increasing laser fluence, finding optimal wavelength, knowing the target’s geometry, optimization of the ablation setup, or temporally and spatially bypassing cavitation bubbles are some of the reported strategies that advance NPs production rate up to three orders of magnitude. However, the ambition to increase production toward industrial implementation is the central goal of most research. For further improvements, the automatization of the LSPC process can be a real turning point in the production rate, since this will allow continuous synthesis production while greatly reducing manual work such as refilling the liquid. Moreover, tailoring the temporal pulse of ultrashort pulses, other than double-pulsed or burst laser irradiation, can change the energy delivery mechanism and influence productivity. Unveiling the fluid dynamics in the ablation chamber can also help to increase productivity by controlling liquid turbulence and bubble removal.^157^

Although PLAL and PLFL brought significant advancements as synthesis techniques for CQDs and GQDs, the lack of study on their practical applications must also be addressed. Therefore, this review also emphasizes the recent developments and progress in implementing laser-synthesized CQDs and GQDs for practical applications. In this article, we showed that they can be a good alternative as bioimaging agent both *in vitro* and *in vivo* owing to their photochemical stability and non-toxicity. Their non-photobleaching and non-photoblinking fluorescence were exploited to develop highly sensitive and selective optical-based fluorescent sensors. Due to their excellent surface composition and reactivity, they also showed great potential as anticancer and antibacterial agents. The potential of laser-synthesized CQDs and GQDs can also be extended to other relevant applications. Due to their facile functionalization with various targeting moieties and therapeutic drugs, they can be used as active or passive targeting fluorescent drug nanocarriers in theranostics. These capabilities will cause a paradigm shift in cancer therapy and diagnosis, particularly in cancer heterogeneity and adaptation. They must also be implemented as drug nanocarriers for cancer, ocular, and infectious diseases. More importantly, their light-harvesting activity over a broad spectral range from UV to near-infrared can be exploited for photothermal, photodynamic cancer therapy, and antibacterial-related applications. More efforts must be made beyond bio-related applications such as wastewater treatment, electrocatalysis, photocatalysis, anticounterfeiting, food packaging and preservation, fingerprint detection, LEDs, and more.

Additionally, CQDs and GQDs as optical-based sensors must be extended to detect and quantify biomolecules, such as nucleic acids, DNA, antibodies, and microorganisms, such as viruses, bacteria, fungi, etc. CQDs and GQDs from other routes were already incorporated into these applications; thus, applying laser-synthesized CQDs and GQDs to these mentioned applications might lead to the discovery of better performance from those synthesized from other routes. In terms of their application itself, there are remaining issues that need further research as well, such as the biological profile evaluation and long-term toxicity of these NPs in humans and the environment, rational design and optimization of their surface chemistry and morphology, and their impact and relationship on biological activity. Engineering structural configurations of CQDs and GQDs to emit fluorescence after excitation by low-energy sources, such as near-infrared, would benefit bioimaging, allowing deep tissue penetration while avoiding background tissue fluorescence. In the field of electrocatalysis, there are few studies regarding the potential of laser-synthesized CQDs and GQDs as electrocatalysts; however, there must be in-depth theoretical and experimental studies of CQDs- and GQDs-based electrocatalysts offering high electrocatalytic ability and long stability.

Laser synthesis in liquids is undeniably an excellent technique to produce NPs; thus, combining it with NPs such as CQDs and GQDs will reroute modern nanotechnology. At their early stage of development, CQDs and GQDs have already shown immense potential in nanotechnology from biorelated (bioimaging, biosensing, drug delivery, antibacterial, anticancer) and beyond bio (electrocatalysis, additives, energy harvesting, forensics, optoelectronics) applications. Although the origin of their fluorescence is not well understood yet, there is no question that they will play a prominent role in inter- and multidisciplinary fields upon further development. Regardless of the shortcomings of the laser synthesis technique, laser-synthesized CQDs and GQDs have an unquestionable ability to provide a comparable or better performance over other synthesis routes, whether in the field of optimization and modification of various properties or the application area. With the power of this technique and the potential of NPs like CQDs and GQDs, nanotechnology and nanoscience are in for a treat. In closing, we hope this review article leads to a better understanding of laser-based nanoparticle synthesis techniques, such as PLAL and PLFL, as tools for fabricating fluorescent CQDs and GQDs. We also hope that this review inspires future research to focus not only on the development of advancing laser synthesis techniques but also on exploring and expanding the applicability of laser-synthesized CQDs and GQDs other than the mentioned applications in this review. We also hope that this review may serve as a reference for future research.
